# Automatic semantic segmentation of the lumbar spine: Clinical applicability in a multi-parametric and multi-center study on magnetic resonance images

**DOI:** 10.1016/j.artmed.2023.102559

**Published:** 2023-06

**Authors:** Jhon Jairo Sáenz-Gamboa, Julio Domenech, Antonio Alonso-Manjarrés, Jon A. Gómez, Maria de la Iglesia-Vayá

**Affiliations:** a FISABIO-CIPF Joint Research Unit in Biomedical Imaging, Fundaciò per al Foment de la Investigaciò Sanitària i Biomèdica (FISABIO), Av. de Catalunya 21, 46020 València, Spain; b Orthopedic Surgery Department, Hospital Arnau de Vilanova, Carrer de San Clemente s/n, 46015, València, Spain; c Radiology Department, Hospital Arnau de Vilanova, Carrer de San Clemente s/n, 46015, València, Spain; d Pattern Recognition and Human Language Technology research center, Universitat Politècnica de València, Camí de Vera, s/n, 46022, València, Spain; e Regional ministry of Universal Health and Public Health in Valencia, Carrer de Misser Mascó 31, 46010 València, Spain

**Keywords:** 92B20, 92C50, 68T07, 68T45, 68U10, 92B10, Magnetic resonance images, Spine, Semantic image segmentation, Convolutional neural networks, Deep learning, Ensembles of classifiers

## Abstract

Significant difficulties in medical image segmentation include the high variability of images caused by their origin (multi-center), the acquisition protocols (multi-parametric), the variability of human anatomy, illness severity, the effect of age and gender, and notable other factors. This work addresses problems associated with the automatic semantic segmentation of lumbar spine magnetic resonance images using convolutional neural networks. We aimed to assign a class label to each pixel of an image, with classes defined by radiologists corresponding to structural elements such as vertebrae, intervertebral discs, nerves, blood vessels, and other tissues. The proposed network topologies represent variants of the U-Net architecture, and we used several complementary blocks to define the variants: three types of convolutional blocks, spatial attention models, deep supervision, and multilevel feature extractor. Here, we describe the topologies and analyze the results of the neural network designs that obtained the most accurate segmentation. Several proposed designs outperform the standard U-Net used as a baseline, primarily when used in ensembles, where the outputs of multiple neural networks are combined according to different strategies.

## Highlights

• State-of-the-art performance in simultaneous segmentation of lumbar spine structures.
• Automatic semantic segmentation of lumbar MR images is achieved using deep learning.
• Complementary blocks into the original U-Net architecture improved its performance.
• Variants of the U-Net architecture were evaluated and combined in ensembles.

## Graphical abstract

**Figure:**
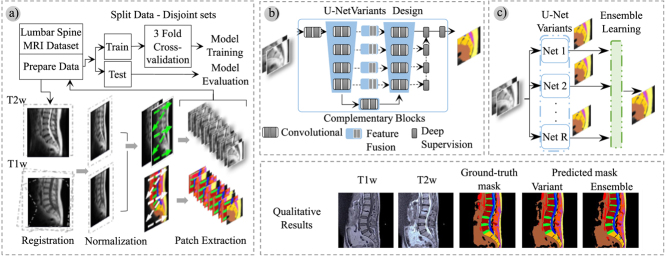

## 1 Introduction

Magnetic resonance (MR) uses magnetic fields with frequencies in the radio wave range (8–130 MHz) to obtain medical images of any part of the human body with an elevated level of detail. MR images provide functional and morphological information on anatomy and pathological processes, with a spatial resolution and contrast higher than those obtained using other medical image acquisition techniques. Concerning lumbar pathologies, MR imaging provides the preferred type of image for radiologists and physicians specialized in the lumbar spine and the spine in general. MR images support the detection of disorders in nerve structures, vertebrae, intervertebral discs, muscles, and ligaments with a high level of precision [1].

### 1.1 Motivation

Manual inspection and analysis by human experts (typically radiologists) represent the most common methodologies to extract information from MR images. Visual inspection is carried out slide by slide to determine the location, size, and pattern of multiple clinical findings (normal or pathological) in lumbar structures. The accurate manual inspection of slides strongly depends on each expert’s experience; therefore, variability introduced due to the different criteria of experts represents a significant challenge [2], [3]. Radiologists, even those with vast experience, require extended periods of time to perform visual inspections of images, a time-consuming and repetitive task. The excess of information that requires visual processing can cause fatigue and loss of attention, prompting the potential loss of perception of specific nuances due to “temporary blindness due to workload excess” [4].

The ongoing development of artificial intelligence (AI) and progress towards its application to medical imaging has provided novel, sophisticated algorithms based on machine learning (ML) techniques. These new algorithms complement existing algorithms in some cases; however, they generally perform significantly better given that most existing algorithms are knowledge-based and do not learn from data. New algorithms provide a much more robust approach to detecting lumbar structures (i.e., vertebrae, intervertebral discs, nerves, blood vessels, muscles, and other tissues) and support a significant reduction in the workload of radiologists and traumatologists [5], [6], [7], [8]. In the context of AI, automatic semantic segmentation currently represents the most widely used technique [9].

The automatic semantic segmentation technique classifies each pixel from an image into one of several classes or categories corresponding to a type of object from the real world to detect. In recent years, convolutional neural networks (CNNs) have been considered the optimal ML technique to address semantic segmentation tasks; however, CNNs require many manually-annotated images to correctly estimate the values of the millions of weights corresponding to all layers of any CNN topology designed by a deep learning (DL) expert. The robustness and precision of any classifier based on CNNs strongly depend on the number of samples available to train the weights of the CNN. Therefore, the challenges in projects addressing the task of semantic segmentation include the availability of a large enough dataset of medical images. To achieve a minimum of samples to train models, ground-truth metadata was obtained by generating bit masks from manual segmentation carried out by two radiologists. Two types of MR images were used – T1 and T2 weighted, T1w and T2w respectively – to manually adjust boundaries between structural elements and tissues. Section 3.1.2 provides more details on both types of MR images.

### 1.2 Aims

The main objective of this study is to use a limited dataset of MR images to accurately and efficiently segment structures and tissues from the lumbar region using individually optimized CNNs or ensembles of several CNNs; we based all topologies on the original U-Net architecture (i.e., U-Net variants).

The proposed work provides a contribution that may guide future research. Mainly, this work: (i) obtains state-of-the-art performance in simultaneous segmentation of lumbar spine structures using DL; (ii) describes the use of complementary blocks in the original U-Net architecture, which improved performance; and (iii) evaluates variants of the U-Net architecture which are combined into ensembles to improve the performance of every single network.

This paper is organized as follows: Section 2 reviews the state-of-the-art and references other studies on the automatic semantic segmentation of medical images. Section 3 details the resources used; Section 3.1 describes the datasets used, and Section 3.2 provides details of the hardware infrastructure and software toolkits used. Section 4 describes the block types employed to design CNN topologies as variants from the original U-Net architecture. Section 5 describes the experiments carried out, Sections 6, 7 present and discuss the results, respectively, and Section 8 concludes by considering the objectives and possible future research.

## 2 Related work

Fully convolutional networks (FCNs) represent one topology of the deep neural networks (DNNs) successfully used for semantic segmentation [10]. FCNs derive from adapting CNNs for image classification and generating a spatial label map as output. FCNs have been compared with AlexNet [11], VGG16 [12], and GoogLeNet [13] by [10]. The topology known as FCN-8, which derives from an adaptation of VGG16, obtained optimal results during the 2012 PASCAL VOC segmentation challenge [14].

Notwithstanding, FCNs suffer from a critical limitation related to semantic segmentation: the fixed size of the receptive field cannot work with objects of different sizes and fragments or misclassifies such objects. Furthermore, relevant details of the objects become lost due to the overly coarse nature of the deconvolution process [15].

Novel approaches have arisen to overpass the limitations of FCNs; however, a subset of approaches derived directly from the FCNs and used deep deconvolution, including SegNet [16], [17] and DeConvnet [15]. SegNet is an autoencoder based on convolutional layers, where each layer in the encoder branch is paired with a layer in the decoder branch (in the sense that they have the same shapes). The *softmax* activation function is used at the output of the last layer of the decoder branch. Adding deeper encoder–decoder layer pairs provides greater spatial context, leading to smoother predictions and improved accuracy when adding more pairs. [18] demonstrated the performance potential of SegNet; their proposed methodology detected lumbar spinal stenosis in axial MR images using semantic segmentation combined with boundary delimitation.

The U-Net network architecture currently obtains the best results [19]. U-Net is an encoder–decoder architecture whose main feature is layer emergence by concatenating features of layers at the same depth (these concatenations are known as skip connections). U-Net has been used with success for semantic segmentation in medical images of the liver [20], kidney [21], skin lesions [22], prostate [23], retinal blood vessels [24], iris [25], brain structures [26], and especially the spine [27], [28], [29], [30].

This work extends our previous study, which focused on segmenting sagittal MR images to delineate structural elements of the anatomy of the lumbar region [31]. There, we analyzed variations of the U-Net architecture by using (a) convolutional blocks [12], [19], (b) spatial attention models [32], (c) deep supervision [33], [34], and (d) multi-kernels at the input, with the latter based on a naive version of the Inception architecture [13]. Integrating these block types improved the performance of the original U-Net architecture; however, not all topologies designed by combining different block types obtained satisfactory results due to the limited size of the dataset sued during experimentation. In our previous study, we used manually annotated MR slides from 75 patients; in this work, we used slides from 181 patients.

Using ensembles of classifiers (combinations of predictive models with similar but different features) represents a widely used strategy to improve the results obtained by classifiers operating alone. In a given ensemble, the combined predictions of several classifiers reduce variance (assuming that the error type of one classifier differs from the other) [35]. Generally, an ensemble possesses better prediction accuracy than the individual classifiers making up the ensemble [36].

[37] reported a comparative study of the performance of four strategies to combine the output of classifiers within ensembles for image recognition tasks. The four strategies were “Unweighted Average” [38], “Majority Voting”, “Bayes Optimal Classifier”, and “Stacked Generalization” [39], [40]. This study reported the use of distinct network structures with different control points and analyzed the problem of overfitting (a typical problem of neural networks) and any impact on ensembles. Other approaches using ensembles in semantic segmentation tasks are based on transfer learning, where networks trained with different datasets from one target task become retrained [41] or on “Stacked U-Nets” trained in two stages. In the latter case, classifier ensembles can detect morphological changes in the cell nucleus by automatically segmenting nuclei regions and regions of overlapping nuclei [42]. The relevance of ensembles has prompted the application of model compression techniques to achieve real-time performance to make predictions in production environments [43].

This work proposes new network topologies derived from the U-Net architecture, representing improvements to previously presented topologies [31]. We obtained the results using individual networks and ensembles combining distinct network topologies. The dataset used to obtain our results represents an extension of the dataset used in our previous work, including manually-segmented MR images from additional patients.

## 3 Resources

**Fig. 1:**
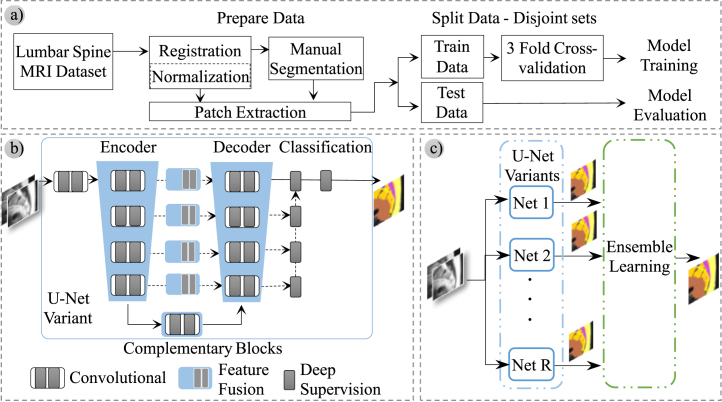
Scheme describing the steps taken in this work: (a) Data preparation and manual segmentation to create ground-truth metadata, (b) Design of the modular framework to define U-Net variants, and (c) Evaluation of individual networks and ensembles to create more sophisticated models by combining different topologies.

Fig. 1 schematically describes the steps followed in this work. In the first step, the lumbar spine MR imaging dataset was selected, processed, and partitioned into two subsets — one for training and validating (corresponding to 80% of patients) and another for testing with images (the remaining 20% of patients). In turn, the first subset was partitioned into two subsets: one to train models (53% of the entire dataset, referred to as the training subset) and another to adjust hyperparameters according to the results obtained (27% of the entire dataset; referred to as the validation subset). This manner of partitioning the most significant subset was repeated three times to obtain three pairs of training and validation subsets to evaluate all models in a three-fold cross-validation procedure.

In the second step, a modular framework was designed to define distinct network topologies derived from the U-Net architecture (each derived topology is the result of combining several complementary and interchangeable blocks). The design and evaluation of distinct topologies were performed in the third and last step, where different configurations of ensembles were also evaluated.

All variants derived from the U-Net architecture have two branches - a descending encoder branch and an ascending decoder branch. Both branches have four levels in all variants evaluated and are connected by a bottleneck block in the deepest level. The classification block is connected to the top layer of the decoder branch and includes the output layer. Predictions from optimal variants were combined using ensemble learning techniques [35], [36], [44]. Section 6 presents the results of individual networks and ensembles, while 4.2 details the different ensembling strategies.

### 3.1 Lumbar spine MR imaging dataset

The MIDAS dataset is an extensive collection of MR images corresponding to the lumbar spine. This dataset represents a primary outcome of the homonym project “Massive Image Data Anatomy of the Spine” (MIDAS). All images from the same scanning session are accompanied by a radiologist’s report (who performed the scan). The MIDAS dataset contains more than 23,600 studies with a total of more than 124,800 MR images. All studies and images correspond to patients who presented with lumbar pathologies during 2015 and 2016 and attended the Valencian Region Health Public System. The public use of the MIDAS dataset was approved by the Ethics committee DGSP-CSISP N^o^ 20190503/12 once all data (images, DICOM metadata, and reports from radiologists) were anonymized correctly by the *“Banco de Imágenes Médicas de la Comunidad Valenciana”* (BIMCV) [45] (https://bimcv.cipf.es/bimcv-projects/project-midas/). Data management and organization, including data curation, followed the standard medical imaging data structure (MIDS) [46].

The dataset used in this work is a subset of the MIDAS dataset, where all selected images were converted from the DICOM format to the NIfTI format, and the reports (together with metadata) were stored using the JSON format. The hierarchical organization of the NIfTI and JSON files follows the same tree structure of MIDS, where all images of a particular scan are located in the same directory, and the directories of all sessions belonging to one patient lie in the same directory at a higher level.

#### 3.1.1 Dataset selection and preparation

The ground-truth dataset for the semantic segmentation task was generated by manually segmenting a subset of the MIDAS dataset obtained by randomly selecting studies corresponding to 181 patients. Each study contains several scanning sessions and several MR images in each session. The selected patients’ ages ranged from 9 to 88 years (with an average of 53 years). The dataset possessed an unbalanced gender distribution, with 105 women and 76 men. The studies used in this work were selected according to the following criteria:

• Lumbar vertebrae and other adjacent anatomical elements must be included, particularly the upper sacral bones
• Each scan should have both types of sagittal MR images available (T1w and T2w), as they will be jointly used as input to the models
• T1w and T2w images from each study must fulfill predefined quality requirements regarding brightness and noise
• Selected patients cannot have undergone lumbar surgery

Due to the different scanning devices used (distinct manufacturers and models), the MR images were acquired with different settings parameters; however, the magnetic field intensity was maintained at 1.5 Teslas in all cases. Table 1 lists the range of values for the relevant configuration parameters according to the metadata accompanying each MR image.

Sagittal T1w and T2w slices from each scanning session were aligned at the pixel level using the FLIRT functionality [47], [48] of the FSL toolkit [49]. The input to the neural networks for every slice is a 3D tensor of H×W×2, where H and W are the height (rows) and the width (columns) of the image in pixels, and 2 is the number of channels. Channel 0 corresponds to T2w, and channel 1 to T1w. Once aligned, both channels’ pixels (T1w and T2w) are normalized to zero mean and unit variance. Normalization is carried out for each channel independently. There were 41,572 MR images in our dataset corresponding to different slices of the lumbar spine area. Most slices have an image resolution of 512 × 512 pixels. The number of slices per scanning session ranges from eight to fourteen.

**Table 1:** Ranges of values of the most relevant configuration parameters of the scan devices.

View plane types	Sagittal	
Sequence types	T1-weighted	T2-weighted
Repetition time (ms)	300.0 to 764.38	2000.0 to 10172.214
Echo time (ms)	6.824 to 17.424	84.544 to 145.0
Spacing between slices (mm)	3.6 to 6.0	3.6 to 6.0
Imaging frequency (MHz)	42.568 to 127.745	42.568 to 127.745
Echo train length	2.0 to 10.0	13.0 to 36.0
Flip angle	80.0 to 160.0	90.0 to 170.0
Height (px)	320.0 to 800.0	320.0 to 1024.0
Width (px)	320.0 to 800.0	320.0 to 1024.0
Pixel spacing (mm)	0.4688 to 1.0	0.3704 to 1.0
Echo number	0.0 to 1.0	0.0 to 1.0

#### 3.1.2 Image labels and ground-truth metadata

The ground-truth metadata for the semantic segmentation task consisted of bit masks generated from the manual segmentation carried out by two radiologists with high expertise in skeletal muscle pathologies.

The ground-truth masks delineate different structures and tissues in a lumbar MR image. The selection of these structures and tissues was carried out by medical consensus, attending to the need of the MIDAS project and regarding the study of the population with a prevalence of lumbar pain, which presents the following radiographic findings: disc dehydration, loss of disc height, disc herniation, Modic changes, facet hypertrophy, yellow ligament hypertrophy, foraminal stenosis, canal stenosis, spondylolisthesis, atrophy of paravertebral musculature and fatty infiltration in the dorsal muscles (thus obtaining the eleven classes of interest).

The input for neural networks comprises T1w and T2w slices aligned at the pixel level. Sagittal T2w images are characterized by highlighting fat and water within the tissues and are used by radiologists to distinguish the anatomical silhouette of the different structural elements of the lumbar region. Sagittal T1w images highlight fat tissue and are used when radiologists have doubts about some anatomical structures or findings (e.g., spinal cavity, epidural fat, or radicular cysts).

**Fig. 2:**
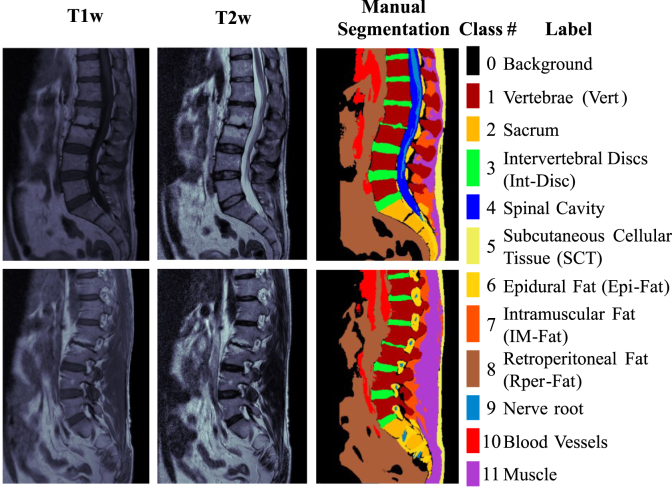
Example of two different slices with corresponding bit masks merged into a single-colored MR image using one different color per class. From left to right: T1w and T2w MR images, ground-truth semantic segmentation, and label summary. (For interpretation of the references to color in this figure legend, the reader is referred to the web version of this article.)

Fig. 2 depicts an example of two different slices from T1w and T2w sagittal images and their semantic segmentation with the labels corresponding to eleven target classes plus the background. The output used to train the neural networks is a stacked 3D tensor containing a one-bit mask per class. In other words, the ground-truth masks are tensors of H×W×12, with twelve values per pixel, with all but one set to 0 (the value corresponding to the class is 1). Fig. 2 represents each class with a different color.

**Fig. 3:**
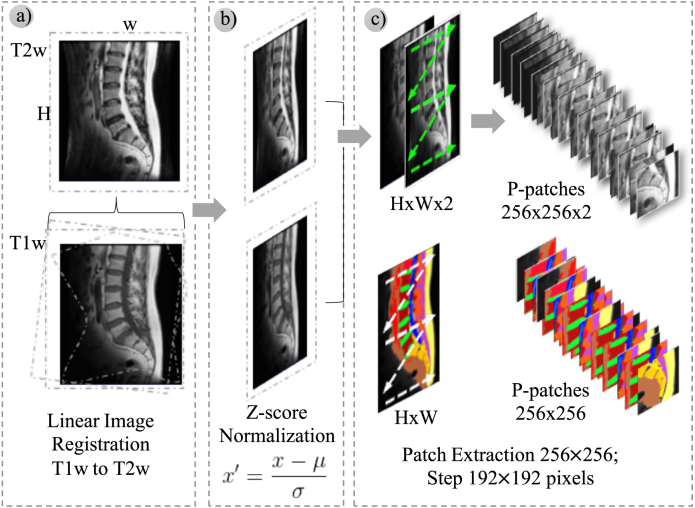
Image preprocessing steps: (a) Linear image registration — sagittal T1w images are aligned with T2w images, (b) Both planes (T1w and T2w) are normalized using the Z-score procedure, (c) Both 2D slices are joined in a 3D tensor of H×W×2, and then, (d) Each 3D tensor and its corresponding ground-truth mask are split into overlapping patches of 256 × 256 pixels.

#### 3.1.3 Patch extraction

As indicated in Section 3.1, image acquisition was carried out using different settings parameters and sizes. The dimension of input samples has relevance when using neural networks, as pixel height and width must be fixed at network input. Resizing all images to a fixed size represents one commonly adopted strategy. The strategy used in this work is different; given an image of H×W pixels, where both H and W can vary from 320 to 1024, squared fragments of fixed size D×D were extracted by shifting approximately S pixels in the horizontal and vertical. An input sample (i.e., a 3D tensor with dimensions H×W×2) is split into overlapping patches with a size of D×D×2 extracted using a stride S×S. Values of D=256 and S=192 were selected based on our previous experimental results [31] to yield a balance between efficiency and accuracy.

Patch extraction was applied to input images and the corresponding ground-truth masks to prepare training and evaluation samples. Ground-truth masks are generated from manual segmentation. Table 2 summarizes the dataset figures, detailing the number of images per partition, the available 2D slices, and the resulting squared fragments or patches. The set of patients in each partition is a disjoint set, i.e., all 2D images (and patches) from one patient lie in the same partition. Fig. 3 depicts the image preprocessing steps and the resulting patches, as explained in Section 3.1.1.

**Table 2:** Dataset used for training and testing in figures.

	Train &		
	Validation	Test	Total
T2w and T1w MR images	148	33	181
2D images	1,176	396	1,572
256 × 256 Patches	18,147	4,113	22,260

### 3.2 Software and hardware

The proposed network topologies were implemented using the TensorFlow [50] and Keras [51] toolkits. The linear (affine) image transformations were carried out using FLIRT [47], [48] from FSL software [49]. The ground-truth masks were manually segmented using ITK-SNAP software [52].

Training and evaluation were carried out using the Artemisa high-performance computing infrastructure from the *“Instituto de Física Corpuscular”* https://artemisa.ific.uv.es (formed by twenty worker nodes equipped with 2 × Intel(R) Xeon(R) Gold 6248 CPU @ 2.50 GHz 20c, 384 GBytes ECC DDR4 at 2933 MHz, 1 × GPU Tesla Volta V100 PCIe). In this system, the complete training of a variant designed from the U-Net architecture following the proposed methodology for 300 epochs requires approximately 12 h in each of the three-fold cross-validation iterations. In the test phase, segmenting one patch of 256 × 256 pixels using the GPU requires less than 0.02s and approximately 10 s using the CPU alone.

## 4 Methodology

### 4.1 Topologies based on the U-Net architecture

**Fig. 4:**
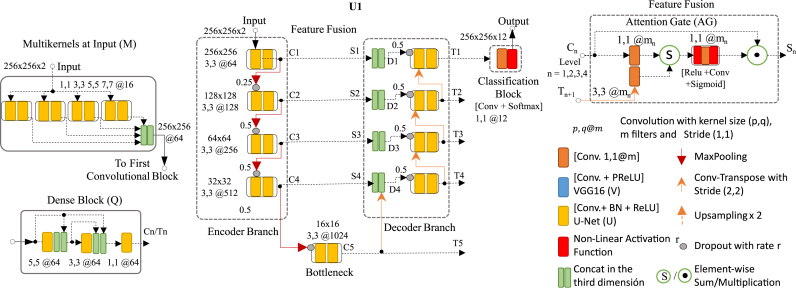
Example of how proposed topologies based on the U-Net architecture (referred to with the identifier U1 in this document) are built from complementary block types: (a) Multi-kernels at the input (M), (b) three types of convolutional blocks (U-net (U), VGG16 (V) and Dense Block (Q)), where U and Q are used in both encoder and decoder branches while V is used only in the encoder branch, (c) Attention Gates (AG) to replace skip connections between encoder and decoder branches to fuse and select relevant features at each level between both branches and (d) Deep supervision (illustrated in Fig. 5). (For interpretation of the references to color in this figure legend, the reader is referred to the web version of this article.)

Different topologies were designed based on the U-Net architecture with the original U-Net architecture used to obtain baseline results. As a first step, a set of distinct interchangeable block types strategically combined to form encoder and decoder branches were defined. Specific topologies presented were designed using different block types in the decoder and encoder branches, while other topologies use the same block type in both branches. Fig. 4 illustrates an example of a U-Net architecture variant and the block types used in the distinct parts of the topology. The following subsections will explain all block types employed.

#### 4.1.1 Convolutional blocks

Three types of convolutional blocks were tested: (i) The typical block used in the original U-Net [19], which consists of two convolutional layers preceding a batch normalization layer followed by an activation layer using the “Rectified Linear Unit” (ReLU). The size of the kernel for both convolutional layers is 3 × 3. (ii) The convolutional block of the VGG16 [12], which is composed of two or three convolutional layers with a 3 × 3 kernel and followed by an activation layer with “Parametric Rectified Linear Unit” (PReLU). (iii) The convolutional dense block [26], which consists of three convolutional layers. A pair of consecutive layers precede each convolutional layer of this block type: a batch normalization layer followed by an activation layer using the “Rectified Linear Unit” (ReLU). The kernel sizes for these three convolutional layers are 5 × 5, 3 × 3 and 1 × 1. The number of channels for the three layers is set to 64. The input to the second layer is the concatenation of the input to the first layer and the output of the first layer. The input to the third layer is the concatenation of the input to the first layer, the output of the first layer, and the output of the second layer. [53] refer to this type of connection as a dense connection.

As Fig. 4 shows, the number of filters (or channels) per block is given by the parameter m at the first (or top) level of the encoder branch (i.e., the descending path); m is multiplied by two when descending from one level to the next (except in the case of the convolutional dense block type, which was set to 64 for all levels). Analogously, m is divided by two when ascending from one level to the next in the decoder branch (i.e., the ascending path).

#### 4.1.2 Multi-kernels at input

The input layer is connected to a multilevel feature extractor in four proposed topologies rather than using only one convolutional block. The multilevel feature extractor consists of four independent convolutional blocks with different kernel sizes (1 × 1, 3 × 3, 5 × 5 and 7 × 7). The output of the four convolutional blocks becomes concatenated before entering the encoder branch to extract spatial features at different scales. This is a variant of the naive version of the Inception network [13].

#### 4.1.3 Encoder branch

The encoder branch is made up of four consecutive convolutional blocks. Each block is followed by a 2D pooling layer with kernel and stride size equal to 2 × 2 to shrink the feature maps to 14 in terms of features (rows and columns divided by 2) while maintaining the depth (number of channels).

#### 4.1.4 Feature fusion

Three strategies of feature fusion were evaluated in this work.

**Fig. 5:**
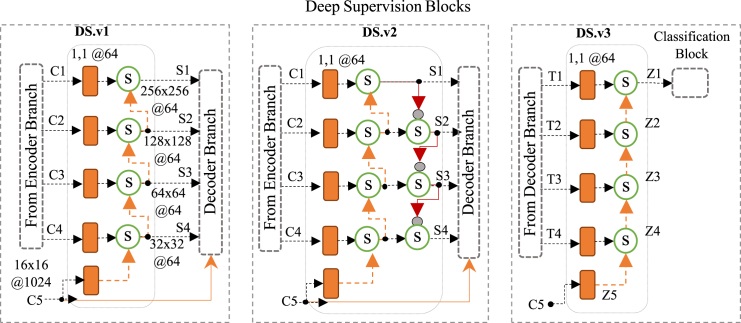
Deep supervision block types. DS.v1 and DS.v2 are alternatives to enhance the connections between the encoder and decoder branches. DS.v3 is used to enrich the input to the classification block; the output of the convolutional block at each level of the decoder branch is combined with an element-wise sum with the supervised signals coming from the previous level of the decoder branch.

(i) The skip connections used in the original U-Net architecture connect blocks at the same level between encoder and decoder branches, represent the first strategy. Feature maps $C_{n}$ from level n in the encoder branch are concatenated with the feature maps $T_{n+1}$ from the previous level in the decoder branch. This is shown in Fig. 4 where $S_{n}=C_{n}$ and $D_{n}=concat\left(S_{n},transposed\text{\_}conv\left(T_{n+1}\right)\right)$ is the input to the convolutional block at level “n” in the decoder branch. The bottleneck output is the special case when $T_{5}=C_{5}$.
(ii) Deep Supervision represents the second strategy, whose underlying idea is to provide a complementary feature-map flow in the decoder branch. Three versions were employed; DS.v1 and DS.v2 are variants of deep supervision used to generate complementary input to the convolutional blocks at each level of the decoder branch, while DS.v3 takes the outputs from the convolutional blocks of the decoder branch to generate a complementary input to the classification block. Deep supervision was introduced by [54] to perform semantic discrimination at different scales in the intermediate layers and as a strategy to mitigate the gradient vanishing problem, as shown by [13] in GoogleNet and [55], [56] in DeepID3. DS.v1 (graphically illustrated in Fig. 5) is proposed as a deep supervision block to replace the skip connections between the encoder and decoder branches. Block type DS.v1 is similar to the block used in DeepID3 by [33], [55], [56] for the same purpose. In more detail, at each level n of the encoder branch (including the bottleneck), the convolutional block generates a feature map (referred to as $C_{n}$) that is transformed by a convolutional layer with a 1 × 1 kernel with m channels, where m is the original number of channels at the first level of the encoder branch. The output tensor at the bottleneck level (i.e., the feature map used to start the decoder branch) is referred to as $C_{5}$ in Fig. 5. The output of the convolutional blocks at each level of the encoder branch is called $C_{n}$. When deep supervision is used, all $C_{n}$ are transformed by a convolutional layer with a 1 × 1 kernel before being combined with the “supervised signal” $S_{n+1}$ coming from the previous level. In DS.v1, the supervised signals are computed as $S_{n}=conv_{1\times 1}\left(C_{n}\right)+up\text{\_}sampling\left(S_{n+1}\right)$, with the especial case of $S_{5}=conv_{1\times 1}\left(C_{5}\right)$. Each $S_{n}$ is concatenated with $transposed\text{\_}conv\left(T_{n+1}\right)$ (i.e., the output of the convolutional block from the previous level in the decoder branch), $T_{n+1}$, is transformed by a transposed convolutional before being concatenated with $S_{n}$ to obtain the input to the convolutional block at level n of the decoder branch: $D_{n}=concat\left(S_{n},transposed\text{\_}conv\left(T_{n+1}\right)\right)$, as in the case of the original U-Net described above. A second deep supervision block type (DS.v2) (see Fig. 5) is used between the encoder and decoder branches. The output of each DS.v2 block at each level is downsampled by a maximum pooling layer with kernel and stride size equal to 2 × 2 to shrink the feature maps to 14 in terms of features (rows and columns divided by 2) while keeping the depth (number of channels) unchanged. In DS.v2, the output of a DS.v2 block (i.e., the supervised signal) at one level is the result of combining the intermediate signal from the lower level and the output of the DS.v2 block from the upper level: $S_{n}=conv_{1\times 1}\left(C_{n}\right)+up\text{\_}sampling\left(prevS_{n+1}\right)+max\text{\_}pool\left(S_{n-1}\right)$, where $prevS_{n+1}$ corresponds to the intermediate signal of the lower level and is calculated as: $prevS_{n+1}=conv_{1\times 1}\left(C_{n+1}\right)+up\text{\_}sampling\left(prevS_{n+2}\right)$. For the sake of ease of understanding, let us highlight that in the U-Net architecture, if we focus our attention on level n, the upper level is n−1 and the lower level is n+1, that could seem contradictory. The special cases are leves 1 and 5, where $S_{1}=prevS_{1}$ and $prevS_{5}=conv_{1\times 1}\left(C_{5}\right)$. One additional deep supervision block type (DS.v3) is used to enrich the input to the classification block. Fig. 5 illustrates how the output of the convolutional blocks at each level of the decoder branch ($T_{n}$) combine with “supervised signals” coming from the previous level, $Z_{n+1}$. The supervised signals are upsampled to achieve the same size of $T_{n}$ to compute the element-wise sum: $Z_{n}=conv_{1\times 1}\left(T_{n}\right)+up\text{\_}sampling\left(Z_{n+1}\right)$, with $Z_{1}$ the input to the classification block in this case. The DS.v3 block type was also used in our previous research for the same purpose: deep supervision [31].
(iii) Attention gate (AG). In the three topologies proposed in this work, the skip connections between the encoder and decoder branches are replaced by a spatial attention model known as the AG [32]. The AG fuses and selects relevant features at each level between both branches; in this manner, the relevant features automatically selected by the AG from the encoder branch are provided to the corresponding level of the decoder branch. With this strategy, the various levels of the decoder branch can use the relevant features extracted at its paired level in the encoder branch for the progressive reconstruction of the output mask. AGs only hold relevant features from the encoder branch that are concatenated with the feature maps obtained as the output of each level in the decoder branch. The feature maps from encoder and decoder branches are transformed individually by a single convolutional layer with a 1 × 1 kernel, which is then combined with an element-wise add operator and passed through a ReLU activation layer followed by another 1 × 1 convolutional layer that, in turn, is followed by a sigmoid activation layer. Sigmoid output values within the range [0,1] function as a 2D mask to filter the feature map from the encoder branch’s respective level. Then, both the AG output $S_{n}$ and the feature map from the previous level of the decoder $T_{n+1}$ are concatenated to connect blocks at the same level; as explained previously $D_{n}=concat\left(S_{n},transposed\text{\_}conv\left(T_{n+1}\right)\right)$. The transposed convolutional resizes $T_{n+1}$ to reach the same size as $S_{n}$. Transposed convolutional layers are represented in orange arrows in Fig. 4.

#### 4.1.5 Bottleneck

The bottleneck is a convolutional block that performs feature estimation at an additional depth level and represents the leading union point between the encoder and decoder branches.

#### 4.1.6 Decoder branch

The decoder branch consists of a set of four consecutive convolutional blocks, with each preceded by a feature-fusion block so that each level of the decoder branch employs the set of relevant features obtained by fusing both (a) the output of the paired convolutional block in the encoder branch with (b) the output of the transposed convolutional layer in the previous level of the decoder branch.

Transposed convolutional layers can better reconstruct the spatial dimension of feature maps in the decoder branch than performing interpolation using an upsampling layer followed by a normal convolution. Transposed convolutional layers can learn a set of weights to reconstruct original inputs progressively. The use of transposed convolutional layers remains crucial when segmenting small structural elements.

#### 4.1.7 Classification block

The output generated by the last level of the decoder branch, or the last level of the deep supervision block (DS.v3), when applicable, is used as input to the classification block. This block consists of one convolutional layer with a 1 × 1 kernel and as many channels as classes to classify each pixel. In our case, the number of classes is twelve. The *softmax* activation function was used at the output layer of all evaluated topologies. The output values are *a posteriori* probability normalized over the predicted output classes. For every pixel of the output mask, each class is weighted by a score in the range [0,1], and the sum of the scores of all classes for a single pixel sums 1. Accordingly, the ground-truth masks used to train the networks have twelve channels, so every pixel of the output mask is represented by one 1-hot vector of length 12. For each pixel of the ground-truth mask, only one of the channels is set to 1.

### 4.2 Ensembles

**Fig. 6:**
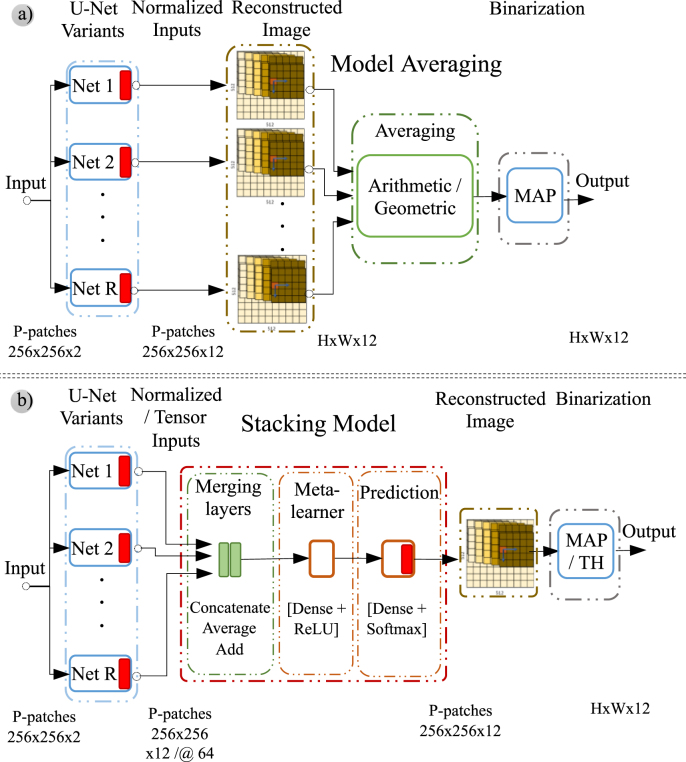
Block diagram of methods evaluated to compute the output of ensembles. (a): Model averaging. (b): Stacking model.

In addition to testing with individual networks, every proposed topology as variants from the U-Net architecture for the semantic segmentation task was evaluated in ensembles of several networks. The outputs of several networks corresponding to different topologies are combined to form a classifier that represents an ensemble of classifiers. The network that obtained the best results was selected from each topology, i.e., the network adjusted with the best combination of hyperparameter values. When used in ensembles, the outputs of single classifiers were combined by two distinct approaches: model averaging and using stacking model. Fig. 6 illustrates both approaches.

#### 4.2.1 Model averaging

Model averaging is a technique where R models equally contribute to obtaining the ensemble’s output, i.e., the prediction provided by the ensemble represents the combination of the prediction of every single model.

Two strategies can be used to merge the outputs of several models:

(1) $$\text{Arithmetic Mean:}\bar{Z}=\frac{1}{R}\overset{R}{\underset{r=1}{\sum }}Z_{r}$$

(2) $$\text{Geometric Mean:}\bar{Z}=\sqrt[{R}]{\overset{R}{\underset{r=1}{\prod }}Z_{r}}$$

#### 4.2.2 Stacking model

Stacking models learn to obtain a better combination of the predictions of R single models to achieve the best prediction. An ensemble following the stacking model is implemented in three stages: (a) *layer merging*, (b) *meta-learner*, and (c) *prediction*.

The first stage, *layer merging*, takes a list of tensors as input and returns a unique tensor that results from concatenating, averaging, or adding. The tensors merged come from every single model in the ensemble and can represent normalized output values (i.e., the output of the *softmax*) or the tensors used as input to the classification block (i.e., the outputs generated by the last level of the decoder branch or DS.v3, where applicable). In the second stage, a dense layer with a ReLU activation function plays the role of *meta-learner*. The last stage - *prediction* - comprises a dense layer with the *softmax* activation function.

### 4.3 Image reconstruction and pixel level labeling

The P patches corresponding to an original 2D slice of size H×W are placed in corresponding positions. Each pixel of the reconstructed mask can belong to 1, 2, or 4 patches. In the case of overlapping (i.e., 2 or 4 patches), the score of each target class per pixel is calculated by using the arithmetic mean of the occurrences of the respective pixel in the overlapping patches. Then, every pixel is labeled with one class according to the following two methods.

#### 4.3.1 Maximum a posteriori probability estimate

The output of the *softmax* activation function in the classification block represents a vector of normalized scores, $y^{m,n}\in R^{12}$, for each single pixel $X^{m,n}$, where X refers to the input image. The element $y_{c}^{m,n}$ is the network’s confidence that pixel $X^{m,n}$ belongs to class c. According to the maximum a posteriori probability (MAP) criterion, every pixel is assigned to the class $c^{*}$ with the highest score, i.e., $c^{*}={\mathrm{argmax}}_{c}\left\{y_{c}^{m,n}\right\}$.

#### 4.3.2 Threshold optimization (TH)

A naive adaptation of the threshold optimization (TH) strategy explained in [57] was used in this work. A threshold per target class was tuned using the validation subset of the three partitions created to carry out the three-fold cross-validation procedure. Section 3 explains how the dataset was partitioned. The threshold of each class was adjusted by finding the maximum value of the intersection over union (IoU) metric for different thresholds ranging from 0.05 to 0.95 using 0.05 increments.

Every pixel at the output is assigned to the class with the highest score generated by the *softmax* activation function if such a score is greater than the threshold for such a class. Otherwise, the score of the following best-scoring class is assessed until a class score greater than or equal to its respective threshold is found. Classes are evaluated in descending order according to score. The pixel is assigned to the background class if this process ends unsuccessfully. MAP or TH will be suffixed to the identifier of each experiment to indicate the method used for labeling every pixel.

## 5 Experiments and implementation

We extracted the dataset used in this work from the MIDAS corpus referenced in Section 3.1. The MR images derive from scanning sessions corresponding to 181 patients, each with a different number of slices (Section 3 explains how we partitioned the dataset into training, validation, and test subsets). Of note, all generated subsets are disjoint at the level of the patient, i.e., no 2D images from the same patient appear in different subsets. Table 2 summarizes the dataset figures. The experiments for each evaluated network topology or ensemble followed the same three-fold cross-validation procedure.

As explained in Section 3, we used 80% of patients for training and validation and 20% for testing. In turn, we split 80% of patients for training and validation into three different partitions to perform a three-fold cross-validation procedure. In each cross-validation iteration, we used images from 23 and 13 of the patients for training and validation, respectively. We obtained the reported results with the test subset as an average of those obtained by the three model versions (one per cross-validation iteration).

We then computed the reported results after labeling every pixel with MAP and TH criteria (see Section 4.3).

### 5.1 Data augmentation

To mitigate the overfitting problem, we randomly modified the training data via the combination of several 2D image transformations: (a) random rotation up to ±20 degrees, (b) zoom in/out by a factor randomly selected from 0.5 to 1.5, (c) random shift in both axes up to 10% of height and width, and (d) horizontal flip according to a Bernoulli probability distribution with p=12.

### 5.2 Model hyper-parameters

All proposed topologies but one represent variations from the U-Net architecture. We identify each complementary block with a letter to construct a list of network identifiers:

**A** AGs to replace the skip connections
**D** Deep supervision between encoder and decoder branches to replace the skip connections (DS.v1 and DS.v2) and between convolutional blocks of the decoder branch to provide an alternative input to the classification block (DS.v3)
**M** A previous step after the input is added before the first block of the encoder branch; several convolutional layers define this step with different kernel sizes whose outputs are concatenated (see Section 4.1.2)
**V** Use of VGG16-like convolutional blocks in the encoder branch (i.e., the descending path); these convolutional blocks are also connected with the convolutional blocks of the decoder branch
**U** The typical convolutional block used in the original U-Net
**Q** Convolutional blocks with dense connections (dense block) to replace U-Net convolutional blocks

Table 3 describes the combination of configuration parameters used to obtain optimal results for each network topology. We trained and evaluated all topologies listed in Table 3 with different combinations of the optimizer, learning rate, and activation function of the hidden convolutional layers (ReLU or PReLU), with the same initial number of channels fixed to 64. In all cases, the activation function of the output layer was the *softmax* value, and the categorical cross-entropy was used as the loss function. In this document, we report the results of only a few topologies and ensembles; the Supplementary Material report the results of all listed topologies. For brevity, we also excluded those designed topologies and combinations of configuration parameters that obtained poor results.

The two variants that involved VGG16 do not use transfer learning, i.e., we estimated the weights of the VGG16 from scratch. In other words, we have not used transfer learning in any of the designed and evaluated topologies. We evaluated the standard U-Net and the FCN to gain baseline results.

**Table 3:** Parameter settings of the CNN topologies. Network IDs are also used in Table 4, Table 6. DS.v2 is only used in topology UDD2.

ID	Configuration	Optimizer	Learning rate	Act-Conv
UDD2	U-Net + DS.v3 + DS.v2	Adam	0.00033	ReLU
UMDD	U-Net + multi-kernel + DS.v3 + DS.v1	Adam	0.00033	ReLU
UDD	U-Net + DS.v3 + DS.v1	Adam	0.00033	ReLU
UQD	U-Net + DenseBlock + DS.v3	Adam	0.00033	ReLU
UVDD	U-Net + VGG16 + DS.v3 + DS.v1	Adam	0.00033	PReLU
UVMD	U-Net + VGG16 + multi-kernel + DS.v3	Adam	0.00033	ReLU
UAMD	U-Net + attGate + multi-kernel + DS.v3	Adam	0.00033	ReLU
UMD	U-Net + multi-kernel + DS.v3	Adam	0.00033	ReLU
UAD	U-Net + attGate + DS.v3	RMSprop	0.001	ReLU
UD	U-Net + DS.v3	Adam	0.00033	ReLU
UA	U-Net + attGate	Adam	0.00033	ReLU
U1	U-Net	Adadelta	1.0	ReLU
FCN	FCN8	Adam	0.00033	ReLU

### 5.3 Model training

We trained all variations designed from the U-Net architecture for 300 epochs using the training subset in the three-fold cross-validation iterations. The optimal version of each model at each cross-validation iteration corresponds to the weight values of the epoch in which the model achieved the highest accuracy with the validation subset.

### 5.4 Ensembles

In addition to training and evaluating individual semantic segmentation models designed as variations from the U-Net architecture, we created a set of ensembles in groups of four to thirteen models. Table 4 reports all ensembles used; note that we used the FCN network only in ensembles E8 and E13.

We performed a dual evaluation approach to compare the two strategies used in ensembles: model averaging and the stacking model. Additionally, we compared results with the arithmetic mean (1) and the geometric mean (2) in the case of model averaging. Fig. 6 depicts the schemes followed in both model averaging and stacking model techniques.

**Table 4:** Abbreviations for the ensembles used and the network identifiers that constitute each ensemble.

Ensemble Id	Networks (IDs) Included
E4	UAD UMD UQD UDD
E5	UD UAD UMD UAMD UDD2
E6	UD UAD UMD UAMD UVMD UVDD
E7	UD UAD UMD UAMD UVMD UQD UDD2
E8	FCN UD UAD UMD UAMD UVMD UQD UDD2
E9	UD UAD UMD UAMD UVMD UVDD UQD UDD UMDD
E10	UD UAD UMD UAMD UVMD UVDD UQD UDD UMDD UDD2
E11	U1 UA UD UAD UMD UAMD UVMD UVDD UQD UDD UMDD
E12	U1 UA UD UAD UMD UAMD UVMD UVDD UQD UDD UMDD UDD2
E13	FCN U1 UA UD UAD UMD UAMD UVMD UVDD UQD UDD UMDD UDD2

Let R be the number of models in an ensemble, let $y_{r}\in R^{12}$ be the output of model r for every pixel with one score $y_{r,c}$ per class (our semantic segmentation task targets twelve classes), and $y\in R^{12}$ be the output of the ensemble per pixel. As all models use the *softmax* activation function in the output layer, their outputs can be normalized and summed to 1, i.e., $\sum_{c}y_{r,c}=1$ and $\sum_{c}y_{c}=1$. Therefore, we consider $y_{r}$ and y as vectors of posterior probabilities and refer to these values as vectors of normalized scores.

The model averaging technique computes the score of each class $y_{c}$ as either the arithmetic mean (1) or the geometric mean (2) from $y_{r,c}\forall r\in \left[1..R\right]$.

We used the stacking model technique with two different approaches to preparing the input to the layer-merging stage: (a) the output of the *softmax* activation layer from each model r in the ensemble, i.e., the vector $y_{r}$, and (b) the 64-channel tensor at the input to the classification block, i.e., the output generated by the last level of the decoder branch or the last level of the deep supervision block (DS.v3) when applicable. Combining the inputs in the layer-merging stage can be carried out by concatenation, averaging, or adding. When the inputs to the ensemble are ready, the two dense layers of the stacking model are trained (see Fig. 6). The ensemble’s output also represents one vector of normalized scores per pixel $y\in R^{12}$.

Table 5 depicts the best-performing ensemble input formats and layer configurations based on the stacking model assembling technique. A three-letter acronym identifies ensemble configurations. The first letter identifies the input type, **N** or **T**, which are normalized scores (*softmax* output) and 64-channel tensors, respectively. The second letter indicates the layer merging operator, averaging (**A**), and concatenation (**C**). We also used the addition operator in the experimentation; however, we do not present the results given their poor quality. The third letter corresponds to the type of meta-learner used; in this case, we only used dense layers with the third letter fixed to **D**.

We trained ensembles based on the stacking model for 50 epochs using the same data-augmentation transformations used to train every single network (see Section 5.1) following the three-fold cross-validation procedure with the same dataset partitions. The optimal version of each stacking model at each cross-validation iteration corresponds to the weight values of the epoch in which the stacking model achieved the highest accuracy with the validation subset.

In both assembling strategies (model averaging and the stacking model), the output masks corresponding to 256 × 256 patches combine and generate a single mask per original slide (medical image) to evaluate the quality of the automatic semantic segmentation. According to the procedure followed to generate the patches from one slice, every pixel of the reconstructed mask can belong to one, two, or four patches. In the case of two or four patches, we used the arithmetic mean to compute the score of each class within the vector of scores of every pixel.

**Table 5:** Parameter settings of optimally-performing stacking models.

Stacking model ID	Configuration				
	Input	Merging layers	Meta-learner	Optimizer	Learning rate
NAD	Normalized	Average	Dense layer	Adam	0.00033
TCD	Tensor	Concatenate	Dense layer	Adam	0.00033

We use the vector corresponding to each pixel of the reconstructed mask to assign each pixel to one of the twelve classes using MAP or TH (see Section 4.3), which we used to evaluate all single networks and ensembles.

### 5.5 Evaluation metrics

We used the IoU metric [10] to compare the performance of network architectures. IoU represents a variant of the Jaccard index used to quantify the overlap between the ground-truth and predicted masks. The IoU for each class c is defined as follows:

(3) $$IoU_{c}=\frac{m_{cc}}{t_{c}+m_{c}-m_{cc}}=\frac{TP_{c}}{TP_{c}+FP_{c}+FN_{c}}$$

where $m_{cc}$ is the count of pixels of class c correctly predicted by the model into the class c, $t_{c}$ is the total amount of pixels of class c according to the ground-truth, and $m_{c}$ is the total amount of pixels assigned to class c by the model. $TP_{c}$ (True Positive of class) is the number of pixels correctly identified as belonging to a specific class, $FP_{c}$ (False Positive of class) is the number of pixels incorrectly identified as belonging to a specific class, and $FN_{c}$ (False Negative of class) is the number of pixels belonging to a specific class that were not identified by the model.

The global metric reported in the results represents the average for all target classes, i.e., all classes except the background class. The averaged IoU can be computed according to the following formula:

(4) $$IoU=\frac{1}{\left|C^{*}\right|}\underset{c\in C^{*}}{\sum }IoU_{c}$$

where $C^{*}$ is the set of classes excluding the background class, i.e., the set of target classes corresponding to each structural element to detect and delimit. The value of IoU varies between 0 and 1, where a value closer to 1 indicates a better accuracy of the model in semantic segmentation for a specific class.

Furthermore, the performance of network architectures are also evaluated based on recall, precision, and the F1-score at the pixel level, which are complementary metrics to IoU and defined as follows:

(5) $$Precision_{c}=\frac{TP_{c}}{TP_{c}+FP_{c}}$$

(6) $$Recall_{c}=\frac{TP_{c}}{TP_{c}+FN_{c}}$$

(7) $$F1-score_{c}=\frac{2TP_{c}}{2TP_{c}+FP_{c}+FN_{c}}$$

## 6 Results

In this work, we addressed the automatic semantic segmentation of lumbar spine MR images using CNNs through single networks and combining the segmentations generated by several networks within ensembles. We aimed to detect and delimit regions in images corresponding to twelve different classes: eleven target classes plus background.

We employed the two criteria described in Section 4.3 to label each pixel into a target class. Using the MAP estimate as the first criterion assigns each pixel at the output to the class with the highest score generated by the *softmax* activation function. Using a naive adaptation of TH as the second criterion, we tuned a threshold per target class using the validation subset to compute the value of the IoU metric for different thresholds. The threshold used for each target class is the one that obtained the best performance.

First, we present a summary of the design of the topologies presented and evaluated. Fig. 4 displays a diagram of the U-Net architecture (U1) used as a baseline and the complementary blocks as an enhancement. We designed all topologies (except those used as a baseline) as variations from the U-Net by strategically using one or more complementary blocks.

Table 3 lists the topologies evaluated and their respective configuration parameters; we present the results of only those obtaining the highest accuracies for brevity (one variant of single networks and four ensembles). The network architectures U1 and FCN correspond to the standard U-Net [19] and FCN8 [10] architectures. We employed the results obtained with these two networks as the baseline to compare results obtained with the proposed variations.

Table 4 reports the evaluated ensembles by grouping different topologies designed as variations from the U-Net architecture. The listed ensembles comprise four to thirteen of the designed network topologies. We used the FCN architecture in two ensembles (E8 and E13) for comparative purposes. Table 6 describes the IoU metric per class computed according to Eq. (3) and the averaged IoU calculated according to Eq. (4) for just one topology of single networks (that obtained optimal results) and the four optimally-performing ensembles. We used the results of topologies FCN and U1 as the baseline. For informational purposes, we report the averaged IoU, including the background class, and highlight optimal results for each class in bold.

Specifically, we report the results of U1, UMD, and E10 in two columns to demonstrate the effect of the two labeling criteria (MAP and TH). TH slightly improves the results of MAP in practically all classes; this improvement is particularly evident for the class *Nerve-Root* (the most difficult to detect). In the particular case of ensemble E13, the two columns show no observable differences between the arithmetic mean or the geometric mean; only the classes *Vert* and *Sacrum* demonstrate some difference in favor of the geometric mean. This finding demonstrates that all topologies combined in this ensemble performed similarly. As expected and previously commented, using ensembles leads to more robust and stable semantic segmentations, which agrees with the observed reduction in the variance of the results among the cross-validation iterations.

**Table 6:** Performance of automatic semantic segmentation via several network topologies and ensembles. Some ensembles performed better using model averaging, while others used the stacking model. The IoU metric is used to evaluate the performance of the twelve classes using Eq. (3). The average with/without the background class was computed using Eq. (4) (Note: background is not a target class). Ensemble E13 obtained satisfactory results with both the arithmetic mean and the geometric mean, and ensemble E10 with both MAP and TH labeling criteria.

Class							Best performing ensembles					
#	Id	Baseline			Best variant		Model averaging		Stacking model			
		FCN	U1	U1	UMD	UMD	E13	E13	E10	E10	E11	E12
							Arith	Geo	TCD	TCD	NAD	NAD
		TH	MAP	TH	MAP	TH	MAP	MAP	MAP	TH	MAP	TH
0	Background	91.8%	92.2%	92.3%	92.2%	92.2%	92.6%	92.6%	92.4%	92.5%	92.6%	92.6%
1	Vert	84.1%	86.0%	86.2%	86.1%	86.3%	86.8%	86.9%	86.6%	86.7%	86.9%	87.0%
2	Sacrum	81.0%	84.1%	84.3%	84.4%	84.8%	85.2%	85.3%	84.8%	85.0%	85.1%	85.4%
3	Int-Disc	86.9%	88.7%	88.9%	88.9%	89.1%	89.4%	89.4%	89.1%	89.3%	89.4%	89.5%
4	Spinal-Cavity	72.6%	75.5%	75.8%	75.9%	76.1%	76.8%	76.8%	76.1%	76.5%	76.5%	77.0%
5	SCT	91.8%	92.5%	92.6%	92.6%	92.6%	93.0%	93.0%	92.8%	92.9%	93.0%	93.1%
6	Epi-Fat	54.6%	58.0%	58.3%	58.5%	58.9%	60.0%	60.0%	59.1%	59.4%	59.6%	60.0%
7	IM-Fat	61.1%	63.8%	64.0%	64.2%	64.6%	65.5%	65.5%	64.8%	65.1%	65.4%	65.7%
8	Rper-Fat	69.3%	70.8%	70.8%	70.5%	70.6%	72.0%	72.0%	71.6%	71.6%	71.9%	72.0%
9	Nerve-Root	45.6%	50.9%	51.8%	51.6%	52.3%	53.1%	53.1%	52.0%	52.6%	52.9%	53.3%
10	Blood-Vessels	58.7%	60.8%	61.3%	60.9%	61.3%	63.0%	63.0%	62.3%	62.6%	63.1%	63.3%
11	Muscle	79.4%	80.8%	81.1%	81.0%	81.2%	81.9%	81.9%	81.4%	81.6%	81.9%	82.0%

**IoU** without Bg.		71.4%	73.8%	74.1%	74.0%	74.3%	75.2%	75.2%	74.6%	74.8%	75.1%	75.3%
**IoU** with Bg.		73.1%	75.3%	75.6%	75.6%	75.8%	76.6%	76.6%	76.1%	76.3%	76.5%	76.7%

Topology UMD obtained the optimal results of all the evaluated variants, outperforming the baseline architecture U-Net (U1) for all classes using the two labeling criteria. The ensemble E12+NAD+TH obtained optimal overall results. The TH labeling criterion performed significantly better than MAP for all experiments. Nevertheless, as discussed later, these differences did not possess statistical significance.

Table 7 shows precision, recall and the F1-score at the pixel level and per class computed according to Eqs. (5), (6), (7), respectively. These metrics are complementary to IoU used in the rest of the paper. And Table 7 compares the best performing topology (UMD+TH) and the best performing ensemble (E12+NAD+TH) with the reference network architecture (U1+TH). The best results for each one of the classes have been highlighted in bold. E12+NAD+TH performed best in all three ranking metrics; notably, it excels against UMD+TH and U1+TH in the *Spinal-Cavity*, *SCT*, *Epi-Fat*, *IM-Fat*, *Rper-Fat*, *Nerve-Root* and *Blood-Vessels* classes.

**Fig. 7:**
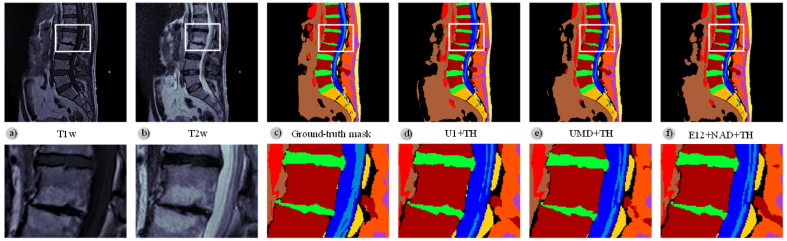
Comparison of the qualitative results of the best-performing topology (UMD+TH) and the best-performing ensemble (E12+NAD+TH) with the baseline network architecture (U1+TH). A zoomed view shows a posterior protrusion of the L1–L2 disc (green - superior) and a marked L2–L3 disc space narrowing (green - inferior). Additionally, the vertebral endplates are affected by Modic changes. This example demonstrates the high quality of the semantic segmentation obtained despite the variability in morphology and signal of the vertebral elements due to the evolution of the pathologies. (For interpretation of the references to color in this figure legend, the reader is referred to the web version of this article.)

Fig. 7 illustrates three examples of predicted masks: one from the best-performing topology (UMD+TH) and another from the best-performing ensemble (E12+NAD+TH) compared with the mask of the baseline architecture (U1+TH). We used the corresponding *T1-weighted* and *T2-weighted* slices as input to the model; Fig. 7 shows the ground-truth mask.

**Fig. 8:**
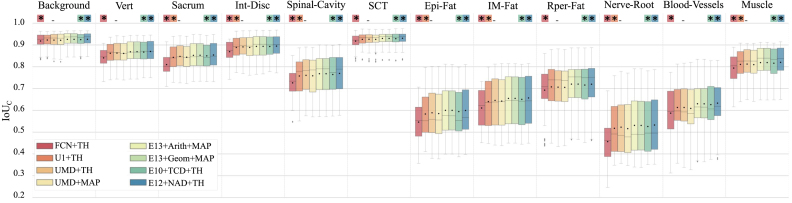
Box plot of intersection over union scores per class, $IoU_{c}$, for comparing UMD+TH (the best variation from the U-Net architecture) with the best ensembles and the two architectures whose results are used as baseline. The 11 target structures in the lumbar region plus the background are represented. 33 MR images from the test subset (split into a total of 396 2D overlapping patches of size 256 × 256) were used for obtaining the classification results to represent the box plots. Same classification results were also used for computing the p-values according to the Wilcoxon signed-rank test in order to check statistical significance of model performance differences. Statistical significance (p<0.05) with respect to UMD+TH is indicated by the star symbol (∗).

**Table 7:** Comparison of the automatic semantic segmentation generated by the best-performing topology (UMD+TH) and the best-performing ensemble (*E12*+NAD+TH) with the reference network architecture (U1+TH) in terms of the metrics precision (5), recall (6) and F1 score (7).

Class		f1-score			Precision			Recall		
#	ID	U1 TH	UMD TH	E12 NAD-TH	U1 TH	UMD TH	E12 NAD TH	U1 TH	UMD TH	E12 NAD TH
0	Background	**96.0%**	**96.0%**	**96.0%**	96.0%	96.0%	**96.7%**	96.0%	96.0%	**96.3%**
1	Vert	**93.0%**	**93.0%**	**93.0%**	92.3%	92.0%	**93.0%**	93.0%	93.3%	**94.0%**
2	Sacrum	91.7%	**92.0%**	**92.0%**	91.3%	**92.0%**	**92.0%**	92.0%	92.0%	**92.3%**
3	Int-Disc	**94.0%**	**94.0%**	**94.0%**	93.0%	**94.0%**	93.3%	**95.0%**	**95.0%**	94.7%
4	Spinal-Cavity	87.0%	87.0%	**87.3%**	**86.7%**	86.0%	**86.7%**	87.0%	88.0%	**88.3%**
5	SCT	96.0%	96.0%	**97.0%**	**97.0%**	96.7%	**97.0%**	96.0%	96.0%	**97.0%**
6	Epi-Fat	73.3%	74.0%	**75.0%**	77.0%	76.7%	**77.3%**	70.3%	71.0%	**71.7%**
7	IM-Fat	78.3%	79.0%	**80.0%**	76.3%	77.3%	**78.3%**	80.0%	80.0%	**80.7%**
8	Rper-Fat	82.7%	82.0%	**83.0%**	81.0%	81.3%	**81.7%**	84.0%	83.3%	**85.0%**
9	Nerve-Root	68.0%	68.0%	**70.0%**	74.3%	76.3%	74.3%	62.3%	61.0%	**65.7%**
10	Blood-Vessels	76.3%	76.0%	**78.0%**	82.7%	82.0%	**83.3%**	70.3%	72.0%	**73.0%**
11	Muscle	90.0%	90.0%	**90.3%**	**91.0%**	**91.0%**	**91.0%**	89.0%	89.0%	**90.0%**

Fig. 8 depicts the box plot of metric $IoU_{c}$ for comparing the topology derived from the U-Net architecture that obtained optimal results (UMD+TH) with the best ensembles and the two architectures whose results we used as a baseline. We used thirty-three MR images from the test subset (split into 396 2D overlapping patches of size 256 × 256) to obtain the classification results to represent the box plots.

We also carried out the Wilcoxon signed-rank test with the same classification results. The null hypothesis $H_{0}$, which can be expressed as *the mean of the difference of each* $IoU_{c}$ *is zero*, is not validated in some cases (using 0.05 as the threshold for the p-value). The results of the two models display statistically significant differences when the p-value exceeds the threshold. We used UMD+TH as the reference model to compute differences. Fig. 8 reports the models that performed differently concerning the UMD+TH model according to the Wilcoxon signed-rank test. Models are highlighted using the star symbol (∗) and independently for each target class.

We can make three observations thanks to the Wilcoxon signed-rank test. Firstly, there existed no significant differences in performance between UMD+TH and UMD+MAP; therefore, we conclude that the TH labeling criterion does not significantly contribute to improvements concerning the MAP criterion based on the test subset used. Notable, the TH labeling criterion depends on adjusting the threshold of each class using a different subset to the test subset. The validation subset adjusted the class-dependent thresholds for all topologies evaluated. There also remains a possibility that this strategy will not provide optimal thresholds for other datasets. Secondly, the UMD+TH performs better than the baseline models. In seven of twelve target classes, UMD+TH performs better than U1+TH, and UMD+TH outperforms FCN+TH in all target classes. Thirdly and most importantly, the ensembles E10+TCD+TH and E12+NAD+TH performed significantly better than UMD+TH for all target classes.

**Fig. 9:**
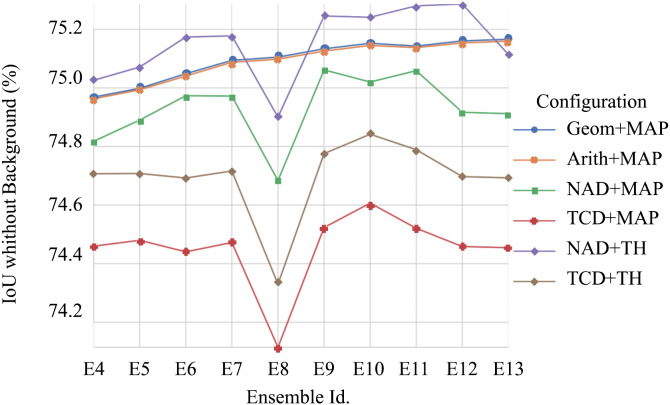
*IoU* metric comparing model averaging and stacking model assembling techniques versus the number of networks in each ensemble.

Fig. 9 compares the assembling techniques employed – model averaging and the stacking model. In the case of model averaging, we considered both means of computing the ensemble’s output from the components’ output – the arithmetic mean and the geometric mean. In the case of the stacking model technique, we considered two-layer merging strategies — averaging and concatenation. Averaging uses the vector of normalized scores at the softmax output, while concatenation uses the input tensors to the classification block.

From Fig. 9, we report the more robust nature of the model averaging assembling technique and the stacking model technique regarding the variance resulting from the predictions of the networks that constitute the ensemble. We did not observe any significant differences between the arithmetic and geometric mean. As mentioned above, the high similarity between both approaches to computing the mean confirms that all topologies combined in the ensembles performed similarly.

Furthermore, Fig. 9 shows that those ensembles, including the FCN topology (E8 and E13), suffer from a significant performance loss when using the stacking model assembling technique. Comparing E12 and E13 results for the configuration NAD+TH demonstrates that adding the FCN topology significantly reduces performance.

We also performed an ablation study to analyze the significance of each component in the optimally-performing ensemble (E12+NAD+TH) to evaluate the impact of each topology derived from the U-Net architecture. We evaluated an ablated ensemble by removing a single model from the ensemble. Table 8 reports the IoU scores for each ablated topology and the difference from the IoU score of the whole ensemble.

In general, removing a single topology (any one of them) has a negative impact on the *Sacrum* and *Cavity-Spinal* classes and a positive impact on the *Nerve-Root* class. In particular, the UMD, UAMD, and UDD topologies contribute to improving the performance of the *Sacrum* and *Cavity-Spinal* classes (removing them has a negative impact), while excluding the U1 or UVDD topologies has a positive impact on the performance of *Nerve-Root* class. All topologies present a similar contribution and removing any of them did not show a significant decrease in overall performance. Obtaining, thus, similar results with the 12 configurations resulting from removing one of the 12 variants of the ensemble, with a minimal penalty for the *Sacrum* and *Cavity-Spinal* classes.

As we could not assess intra- and inter-observer variability in the manual annotation process, we evaluated the best-performing topology (UMD+TH) in a similar task as an alternative strategy. We employed the publicly available Mendeley MRI image database [28]. [27] previously manually labeled axial views of the last three levels of intervertebral discs in 515 scans of subjects with symptomatic back pain. The authors defined the following labels: *intervertebral disc* (IVD), *posterior element* (PE), *thecal sac* (TS) and *area between the anterior and posterior vertebral elements* (AAP), and reported high inter-rater agreement in three classes (IVD, PE, TS). In another study, [18] used the same dataset to segment and detect spinal stenoses using the U-Net architecture in a network topology called SegNet-TL80. To compare these results, we adapted the UMD+TH topology classification block to obtain the four classes plus background. We trained the model for thirty additional epochs in the new axial MR imaging context, employing the three-fold cross-validation procedure and data augmentation method described above. We used the remaining 20% of the data in model evaluation.

**Table 8:** The impact of each component on the overall performance of the best-performing proposed ensemble (E12+NAD+TH). The IoU metric was used to evaluate the performance of the twelve classes using Eq. (3). The average with/without the background class was computed using Eq. (4) (Note: background is not a target class).

Class		Impact E12 NAD TH without :											
#	Id	U1		UA		UD		UAD		UMD		UAMD	
		IoU	Impact	IoU	Impact	IoU	Impact	IoU	Impact	IoU	Impact	IoU	Impact
0	Background	92.6%	–	92.6%	–	92.6%	–	92.6%	–	92.6%	–	92.6%	–
1	Vert	87.0%	–	87.0%	–	87.0%	–	86.9%	−0.001	87.0%	–	86.9%	−0.001
2	Sacrum	85.2%	−0.002	85.3%	−0.001	85.2%	−0.002	85.2%	−0.002	85.2%	− 0.003	85.1%	− 0.003
3	Int-Disc	89.5%	–	89.5%	–	89.5%	–	89.4%	–	89.5%	–	89.5%	–
4	Spinal-Cavity	76.8%	−0.001	76.9%	−0.001	76.8%	−0.001	76.8%	−0.001	76.9%	−0.001	77.0%	–
5	SCT	93.1%	–	93.1%	–	93.0%	–	93.0%	–	93.0%	–	93.1%	–
6	Epi-Fat	60.0%	0.001	60.0%	–	60.0%	0.001	59.9%	–	60.0%	–	59.9%	–
7	IM-Fat	65.7%	–	65.7%	–	65.6%	–	65.7%	–	65.7%	–	65.7%	–
8	Rper-Fat	72.0%	–	72.0%	–	71.9%	–	72.0%	–	72.0%	–	72.0%	–
9	Nerve-Root	53.6%	0.003	53.3%	0.001	53.4%	0.001	53.3%	–	53.4%	0.001	53.3%	–
10	Blood-Vessels	63.3%	–	63.2%	−0.001	63.2%	−0.001	63.4%	0.001	63.4%	0.001	63.4%	0.001
11	Muscle	82.0%	–	82.0%	–	82.0%	–	82.0%	–	82.0%	–	82.0%	–

#	Id	UVMD		UVDD		UQD		UDD		UMDD		UDD2	
		IoU	Impact	IoU	Impact	IoU	Impact	IoU	Impact	IoU	Impact	IoU	Impact

0	Background	92.6%	–	92.6%	–	92.6%	–	92.6%	–	92.6%	–	92.6%	–
1	Vert	87.0%	–	87.0%	–	87.0%	–	87.0%	–	87.0%	–	87.0%	–
2	Sacrum	85.3%	−0.001	85.2%	−0.002	85.3%	−0.001	85.2%	−0.002	85.2%	−0.002	85.4%	–
3	Int-Disc	89.5%	–	89.4%	−0.001	89.5%	–	89.5%	–	89.5%	–	89.5%	–
4	Spinal-Cavity	76.8%	−0.001	76.9%	−0.001	76.8%	−0.001	76.9%	−0.001	76.9%	–	76.8%	−0.001
5	SCT	93.0%	–	93.1%	–	93.1%	–	93.1%	–	93.0%	–	93.0%	–
6	Epi-Fat	60.0%	0.001	59.9%	–	59.9%	–	60.0%	0.001	60.0%	–	59.9%	–
7	IM-Fat	65.7%	–	65.7%	–	65.7%	–	65.7%	–	65.7%	–	65.6%	–
8	Rper-Fat	72.0%	–	72.0%	–	72.0%	–	72.0%	–	72.0%	–	72.0%	–
9	Nerve-Root	53.4%	0.001	53.5%	0.003	53.4%	0.001	53.3%	–	53.4%	0.001	53.3%	0.001
10	Blood-Vessels	63.2%	−0.001	63.2%	−0.001	63.2%	−0.001	63.3%	−0.001	63.4%	0.001	63.4%	0.001
11	Muscle	82.0%	–	82.0%	–	82.0%	–	82.0%	–	82.0%	–	82.1%	–

Table 9 compares the results reported by [18] and those obtained with the UMD+TH topology previously trained on our dataset. Overall, the UMD topology obtained better results, outperforming the reference model Segnet-TL80 in all classes using the TH labeling criterion.

In summary, the variants from the U-Net architecture and, thus, the proposed ensembles outperform the proposed baseline in most classes, suggesting a positive outcome of this research. Our proposed approach demonstrates high performance in the segmentation of clinically relevant structures (e.g., mainly discs, vertebrae, and spinal canal) despite the variability in the quality and provenance of the MR scans.

**Table 9:** Comparison of the performance of automatic semantic segmentation reported by *SegNet-TL80*[18] and generated with UMD+TH. The Intersection over Union (IoU) was the metric used to evaluate the performance of the five classes in common. [18] defined the following labels: *intervertebral disc* (IVD), *posterior element* (PE), *thecal sac* (TS) and *area between the anterior and posterior vertebral elements* (AAP), background (Bg) is not a target class.

SegNet-TL80			UMD+TH		
#	Ax-Label	IoUc	#	Sag-Label	IoUc
0	Bg	98%	0	Background	99.4%
1	IVD	92%	4	Intervertebral disc	96.8%
2	PE	78%	1	Vertebrae	91.2%
3	TS	85%	5	Spinal cavity	90.5%
4	AAP	53%	7	Epidural fat	74.1%

IoU without Bg.		77%			88.2%

## 7 Discussion

Data and metadata played a crucial role in this study. Collecting data represented a critical task that consisted of (i) centralizing MR images from distinct hospitals with corresponding reports generated by radiologists, (ii) revising image quality for each session to identify those with validity, and (iii) anonymizing both images and reports. Generating the ground-truth mask for every image represented the most challenging task. As explained in Section 3.1 and summarized in Table 2, we manually segmented and used only 1.572 images from 181 patients. The ground-truth masks represent the product of the manual semantic segmentation of images to delimit the eleven target classes plus the background from the anatomical components of the lumbar region visible in sagittal T1w and T2w MR images. Each pixel of the ground-truth masks becomes assigned to only one of twelve classes. As mentioned, this work focuses on the lumbar region to automatically delimit anatomical structures and tissues from sagittal MR images. We acquired images from scanning sessions from various hospitals in the Valencian region and corresponded to different pathologies.

### 7.1 Medical perspective

We designed a specific procedure to semantically segment structures and tissues of the lumbar region based on single CNNs and ensembles of CNNs. The procedure performs a multiclass segmentation with promising results in relevant structures from the clinical point of view: *vertebrae, intervertebral discs, spinal cavity, muscle, subcutaneous cellular tissue*, and *intra-muscular fat*.

Notably, the segmentation of relevant structures such as *nerve roots* and *epidural fat* presented a more challenging task (nerve roots appear in sagittal slices as small structures at the level of intervertebral foramen). We achieved $IoU_{c}$ values of 53.3% and 60.0% for nerve roots and epidural fat using the optimal ensemble (E12+NAD+TH), representing very low values compared to other structures. The segmentation quality strongly depends on the size of the object detected; to mitigate this problem, we considered intradural and extradural nerve roots as one class – the target class *Nerve-root*. Despite this decision, we discovered that most errors concerning class *Nerve-root* were false negatives, i.e., pixels corresponding to this class became mislabeled.

Using multi-kernels to process the image at the input layer with receptive fields of different sizes represents one strategy used to cope with the problem of small objects. The output of the convolutional layers with different kernel sizes whose input is the input layer becomes stacked together by concatenation. Topologies UMD, UMDD, UVMD, and UAMD use multi-kernels. [58] used this multiresolution and multi-scale strategy in a coronary vessel segmentation task, obtaining promising results compared to twenty state-of-the-art visual segmentation methods using a benchmark X-ray coronary angiography database.

Analyzing other published studies devoted to the semantic segmentation of brain images [26] suggests that the lumbar spine’s structural complexity compares well to the complexity of the brain. Both cases have many structural elements whose morphology significantly changes between the slices of the same scanning session. The number of slices in scanning sessions of the brain remains much higher; therefore, we consider all images from a scan as a 3D object and rescale said object to an isotropic space with a resolution that each pixel of a 2D image represents an area of around 1 mm^2^. Similar transformations using the images available for this study remain impossible due to the lower number of sagittal slices and the fact that scanning sessions have a different number of slices (i.e., the variance in the distance between sagittal slices remains too high for this purpose). Additionally, more observed variations occur in spinal scans (due to aging and different pathologies) than in available brain scans. Usually, patients with different brain and neurological pathologies possess more similar patterns when compared to patients with distinct spine pathologies. The high range of variations induced by the degeneration of intervertebral discs – common findings in symptomatic and asymptomatic individuals – represents a robust example [59], [60], [61].

### 7.2 Limitations

The following limitations represented critical challenges to this study.

(a) MR images were acquired using distinct models of scanning devices and from different manufacturers that, in addition, were not calibrated in the same manner; hence, acquisition parameters were not homogeneous. To minimize the impact of configuration parameter variability, we selected images based on parameters within specific ranges (Table 1). Despite parameter variability, the quality of the automatic semantic segmentation confirms the robustness of the proposed models and their potential for use by clinicians.
(b) Low image quality due to intrinsic factors of scanning devices, such as sensitivity.
(c) Overlapping and ambiguous elements make assigning classes to such elements challenging, which requires considerable expertise to carry out manual semantic segmentation due to the complexity of anatomical structure. Two radiologists generated the ground-truth metadata; however, the manual segmentation of the images from each scanning session was carried out by just one radiologist due to time constraints. Therefore, we could not compare different manual segmentations of the same images provided by different radiologists. On average, one radiologist took five to eight hours to segment the twelve slices that, on average, come from a single scanning session.
(d) The proposed models were not configured to appreciate tissue patterns and findings not included in the training data (such as tumors and cysts). We assigned all elements encountered during manual segmentation that did not belong to any of the target classes to the background class.

## 8 Conclusions and future works

This work addressed the problem of segmenting sagittal MR images corresponding to the lumbar spine with eleven target classes. Each target class corresponds to one structural element of the lumbar region’s anatomy. We used one additional class (the background class) to help the neural networks distinguish regions of the image that do not correspond to any anatomical structures of interest. We designed eleven network topologies as variations of the U-Net architecture to address the problem and evaluated topologies both individually and combined in ensembles. Considering the results reported here, we achieved the primary objective defined in Section 1.

Several proposed topologies and ensembles of neural networks outperformed both network architectures (the FCN and the original U-Net) used as the baseline. Remarkably, we observed significantly better results of the topology UMD and the ensembles E10+TCD+TH and E12+NAD+TH compared to the results of the baseline architectures according to the Wilcoxon signed-rank test. Moreover, these two ensembles also performed significantly better than the topology UMD according to the same Wilcoxon signed-rank test.

Complementary blocks used to enhance the original U-Net architecture improved performance. The block types used in this work included deep supervision, spatial attention using AGs, multi-kernels at the input, and the VGG16 topology for the encoder branch; however, combining all complementary block types did not provide optimal results. Most variants that included deep supervision in the decoding branch improved the baseline. The Supplementary Material describe all individual topologies evaluated.

Regarding ensembles, all combinations of topologies trained with the predictions of individual topologies and following the three-fold cross-validation procedure with the same partitions of the dataset performed better than any particular topology with the validation subset.

The ensembles based on the averaging-model assembling technique displayed greater robustness to network prediction variance than those based on the stacking-model technique. In the particular case of the ensembles based on the averaging-model technique, we observed marginally better geometric mean results than those obtained using the arithmetic mean; nevertheless, the Wilcoxon signed-range test failed to report this improvement as statistically significant. As mentioned, the two ensembles that provided optimal overall results were based on the stacking model technique.

Intervertebral discs and vertebrae can be easily detected due to the homogeneity of textures and morphology. In our future research, we will focus on the most challenging target classes to improve the quality of automatic semantic segmentation. Nerve roots, epidural fat, intramuscular fat, and blood vessels represent the most challenging classes due to heterogeneity in morphology and textures; furthermore, nerve roots do not appear in the slices with the same frequency as other anatomical structures. The imbalance in the number of samples of the different target classes in the training subset makes the less frequent classes much more difficult to detect, as the model cannot observe sufficient samples (2D images, in this case) containing regions of such classes. Imbalance plus heterogeneity of textures and morphologies make it incredibly challenging to detect some classes more accurately.

## CRediT authorship contribution statement

**Jhon Jairo Sáenz-Gamboa:** Conceptualization, Methodology, Software, Validation, Formal analysis, Investigation, Data curation, Writing – original draft, Writing – review & editing, Final version, Visualization. **Julio Domenech:** Conceptualization, Methodology, Investigation, Resources, Data curation, Review. **Antonio Alonso-Manjarrés:** Conceptualization, Methodology, Investigation, Data curation, Review. **Jon A. Gómez:** Conceptualization, Methodology, Formal analysis, Investigation, Supervision, Funding acquisition, Writing – original draft, Writing – review & editing, Final version. **Maria de la Iglesia-Vayá:** Conceptualization, Methodology, Formal analysis, Resources, Investigation, Supervision, Funding acquisition, Writing – original draft, Writing – review & editing, Final version.

## Declaration of Competing Interest

The authors declare that they have no known competing financial interests or personal relationships that could have appeared to influence the work reported in this paper.

## Appendix A Supplementary materials

MMC S1

## References

[1] Roudsari B., Jarvik J.G. (2010). Lumbar spine MRI for low back pain: indications and yield. Am J Roentgenol.

[2] Carrino J.A., Lurie J.D., Tosteson A.N., Tosteson T.D., Carragee E.J., Kaiser J., Grove M.R., Blood E., Pearson L.H., Weinstein J.N. (2009). Lumbar spine: reliability of MR imaging findings. Radiology.

[3] Berg L., Neckelmann G., Gjertsen Ø., Hellum C., Johnsen L.G., Eide G.E., Espeland A. (2012). Reliability of MRI findings in candidates for lumbar disc prosthesis. Neuroradiology.

[4] Konstantinou N., Bahrami B., Rees G., Lavie N. (2012). Visual short-term memory load reduces retinotopic cortex response to contrast. J Cogn Neurosci.

[5] Coulon O., Hickman S., Parker G., Barker G., Miller D., Arridge S. (2002). Quantification of spinal cord atrophy from magnetic resonance images via a B-spline active surface model. Magn Reson Med: Off J Int Soc Magn Reson Med.

[6] Van Uitert R., Bitter I., Butman J.A. (2005). International congress series, Vol. 1281.

[7] De Leener B., Kadoury S., Cohen-Adad J. (2014). Robust, accurate and fast automatic segmentation of the spinal cord. NeuroImage.

[8] De Leener B., Cohen-Adad J., Kadoury S. (2015). Automatic segmentation of the spinal cord and spinal canal coupled with vertebral labeling. IEEE Trans Med Imaging.

[9] Litjens G., Kooi T., Bejnordi B.E., Setio A.A.A., Ciompi F., Ghafoorian M., van der Laak J.A., van Ginneken B., Sánchez C.I. (2017). A survey on deep learning in medical image analysis. Med Image Anal.

[10] Long J, Shelhamer E, Darrell T. Fully convolutional networks for semantic segmentation. In: 2015 IEEE conference on computer vision and pattern recognition. CVPR, 2015, p. 3431–40. 10.1109/CVPR.2015.7298965. 27244717

[11] Krizhevsky A., Sutskever I., Hinton G.E., Pereira F., Burges C.J.C., Bottou L., Weinberger K.Q. (2012). Advances in neural information processing systems, Vol. 25.

[12] Simonyan K, Zisserman A. Very Deep Convolutional Networks for Large-Scale Image Recognition. In: International conference on learning representations. 2015, p. 1–14, arXiv:1409.1556v6.

[13] Szegedy C, Liu W, Jia Y, Sermanet P, Reed S, Anguelov D, Erhan D, Vanhoucke V, Rabinovich A. Going deeper with convolutions. In: 2015 IEEE conference on computer vision and pattern recognition. CVPR, 2015, p. 1–9. 10.1109/CVPR.2015.7298594.

[14] Everingham M., Van Gool L., Williams C.K., Winn J., Zisserman A. (2010). The pascal visual object classes (voc) challenge. Int J Comput Vis.

[15] Noh H, Hong S, Han B. Learning deconvolution network for semantic segmentation. In: 2015 IEEE international conference on computer vision. ICCV, 2015, p. 1520–8. 10.1109/ICCV.2015.178.

[16] Badrinarayanan V., Handa A., Cipolla R. (2015). http://arxiv.org/abs/1505.07293.

[17] Badrinarayanan V., Kendall A., Cipolla R. (2017). Segnet: A deep convolutional encoder-decoder architecture for image segmentation. IEEE Trans Pattern Anal Mach Intell.

[18] Al-Kafri A.S., Sudirman S., Hussain A., Al-Jumeily D., Natalia F., Meidia H., Afriliana N., Al-Rashdan W., Bashtawi M., Al-Jumaily M. (2019). Boundary delineation of MRI images for lumbar spinal stenosis detection through semantic segmentation using deep neural networks. IEEE Access.

[19] Ronneberger O., Fischer P., Brox T. (2015). Medical image computing and computer-assisted intervention – MICCAI 2015.

[20] Christ P.F., Elshaer M.E.A., Ettlinger F., Tatavarty S., Bickel M., Bilic P., Rempfler M., Armbruster M., Hofmann F., D’Anastasi M. (2016). International conference on medical image computing and computer-assisted intervention.

[21] Çiçek Ö., Abdulkadir A., Lienkamp S.S., Brox T., Ronneberger O. (2016). International conference on medical image computing and computer-assisted intervention.

[22] Lin BS, Michael K, Kalra S, Tizhoosh HR. Skin lesion segmentation: U-nets versus clustering. In: 2017 IEEE symposium series on computational intelligence. SSCI, 2017, p. 1–7. 10.1109/SSCI.2017.8280804.

[23] Yu L, Yang X, Chen H, Qin J, Heng PA. Volumetric ConvNets with mixed residual connections for automated prostate segmentation from 3D MR images. In: Proceedings of the AAAI conference on artificial intelligence, Vol. 31. 2017, p. 66–72. 10.5555/3298239.3298250.

[24] Xiao X., Lian S., Luo Z., Li S. (2018). 2018 9th international conference on information technology in medicine and education.

[25] Lian S., Luo Z., Zhong Z., Lin X., Su S., Li S. (2018). Attention guided U-net for accurate iris segmentation. J Vis Commun Image Represent.

[26] Roy A.G., Conjeti S., Navab N., Wachinger C., Initiative A.D.N. (2019). QuickNAT: A fully convolutional network for quick and accurate segmentation of neuroanatomy. NeuroImage.

[27] Friska N, Hira M, Nunik A, Ala S. A-K, Sud S, Andrew S, Ali S, Mohammed A-J, Wasfi A-R, Mohammad B. Development of Ground Truth Data for Automatic Lumbar Spine MRI Image Segmentation. In: 2018 IEEE 20th international conference on high performance computing and communications; IEEE 16th international conference on smart city; IEEE 4th international conference on data science and systems (HPCC/SmartCity/DSS). 2018, 10.1109/hpcc/smartcity/dss.2018.00239.

[28] Sudirman S., Al Kafri A., Natalia F., Meidia H., Afriliana N., Al-Rashdan W., Bashtawi M., Al-Jumaily M. (2019). Lumbar spine MRI dataset. Data Mendeley Com.

[29] Huang J., Shen H., Wu J., Hu X., Zhu Z., Lv X., Liu Y., Wang Y. (2020). Spine explorer: a deep learning based fully automated program for efficient and reliable quantifications of the vertebrae and discs on sagittal lumbar spine MR images. Spine J.

[30] Li H., Luo H., Huan W., Shi Z., Yan C., Wang L., Mu Y., Liu Y. (2021). Automatic lumbar spinal MRI image segmentation with a multi-scale attention network. Neural Comput Appl.

[31] Saenz-Gamboa JJ, de la Iglesia-Vayá M, Gómez JA. Automatic Semantic Segmentation of Structural Elements related to the Spinal Cord in the Lumbar Region by using Convolutional Neural Networks. In: 2020 25th international conference on pattern recognition. ICPR, 2021, p. 5214–21. 10.1109/ICPR48806.2021.9412934.

[32] Schlemper J., Oktay O., Schaap M., Heinrich M., Kainz B., Glocker B., Rueckert D. (2019). Attention gated networks: Learning to leverage salient regions in medical images. Med Image Anal.

[33] Zeng G., Yang X., Li J., Yu L., Heng P.-A., Zheng G. (2017). International workshop on machine learning in medical imaging.

[34] Goubran M., Ntiri E.E., Akhavein H., Holmes M., Nestor S., Ramirez J., Adamo S., Ozzoude M., Scott C., Gao F. (2020). Hippocampal segmentation for brains with extensive atrophy using three-dimensional convolutional neural networks. Hum Brain Map.

[35] Goodfellow I., Bengio Y., Courville A. (2016). http://www.deeplearningbook.org.

[36] Bishop C.M. (1995).

[37] Ju C., Bibaut A., van der Laan M. (2018). The relative performance of ensemble methods with deep convolutional neural networks for image classification. J Appl Stat.

[38] Breiman L. (2001). Random forests. Mach Learn.

[39] Wolpert D.H. (1992). Stacked generalization. Neural Netw.

[40] Van der Laan M.J., Polley E.C., Hubbard A.E. (2007). Super learner. Statist Appl Genet Mol Biol.

[41] Nigam I., Huang C., Ramanan D. (2018). 2018 IEEE winter conference on applications of computer vision.

[42] Kong Y., Genchev G.Z., Wang X., Zhao H., Lu H. (2020). Nuclear segmentation in histopathological images using two-stage stacked U-nets with attention mechanism. Front Bioeng Biotechnol.

[43] Holliday A., Barekatain M., Laurmaa J., Kandaswamy C., Prendinger H. (2017). Speedup of deep learning ensembles for semantic segmentation using a model compression technique. Comput Vis Image Underst.

[44] Perrone M., Cooper L. (1993). When networks disagree: Ensemble methods for hybrid neural networks. Neural Netw Speech Image Process.

[45] de la Iglesia-Vayá M., Salinas J.M., Rojas G.M., Pérez Cortés J., Llobet R., Cazorla M.Á., Martínez J., Martí-Bonmatí L., Blanquer I., Regaña M. (2014). BIMCV: Synergy between peta bytes of data in population medical imaging, computer aided diagnosis and AVR. Stud Health Technol Inform.

[46] Saborit-Torres J.M., Saenz-Gamboa J.J., Montell J.A., Salinas J.M., Gómez J.A., Stefan I., Caparrós M., García-García F., Domenech J., Manjón J.V., Rojas G., Pertusa A., Bustos A., González G., Galant J., de la Iglesia-Vayá M. (2020). http://arxiv.org/abs/2010.00434.

[47] Jenkinson M., Smith S. (2001). A global optimisation method for robust affine registration of brain images. Med Image Anal.

[48] Jenkinson M., Bannister P., Brady M., Smith S. (2002). Improved optimization for the robust and accurate linear registration and motion correction of brain images. Neuroimage.

[49] Jenkinson M., Beckmann C.F., Behrens T.E., Woolrich M.W., Smith S.M. (2012). Fsl. Neuroimage.

[50] Abadi M, Barham P, Chen J, Chen Z, Davis A, Dean J, Devin M, Ghemawat S, Irving G, Isard M, et al. Tensorflow: A system for large-scale machine learning. In: 12th USENIX symposium on operating systems design and implementation (OSDI’16). 2016, p. 265–83. 10.5555/3026877.3026899.

[51] Chollet F. (2015). https://github.com/fchollet/keras.

[52] Yushkevich P.A., Piven J., Cody Hazlett H., Gimpel Smith R., Ho S., Gee J.C., Gerig G. (2006). User-guided 3D active contour segmentation of anatomical structures: Significantly improved efficiency and reliability. Neuroimage.

[53] Huang G., Liu Z., Van Der Maaten L., Weinberger K.Q. (2017). 2017 IEEE conference on computer vision and pattern recognition.

[54] Lee C.-Y., Xie S., Gallagher P., Zhang Z., Tu Z. (2015). Artificial intelligence and statistics.

[55] Sun Y., Liang D., Wang X., Tang X. (2015). http://arxiv.org/abs/1502.00873.

[56] Shen Z., Liu Z., Li J., Jiang Y.-G., Chen Y., Xue X. (2019). Object detection from scratch with deep supervision. IEEE Trans Pattern Anal Mach Intell.

[57] Lepora N.F. (2016). Proceedings of the 30th international conference on neural information processing systems.

[58] Jiang Z., Ou C., Qian Y., Rehan R., Yong A. (2021). Coronary vessel segmentation using multiresolution and multiscale deep learning. Inform Med Unlocked.

[59] Tehranzadeh J., Andrews C., Wong E. (2000). Lumbar spine imaging: normal variants, imaging pitfalls, and artifacts. Radiol Clin.

[60] Lundon K., Bolton K. (2001). Structure and function of the lumbar intervertebral disk in health, aging, and pathologic conditions. J Orthop Sports Phys Therapy.

[61] Benoist M. (2005). Natural history of the aging spine. Aging Spine.

