# Supplementary material for: Automatic semantic segmentation of the lumbar spine: Clinical applicability in a multi-parametric and multi-center study on magnetic resonance images
Source: Artif Intell Med. 2023 Jun;140:102559. doi: 10.1016/j.artmed.2023.102559 (PMC10242366; doi:10.1016/j.artmed.2023.102559)
Supplement: MMC S1 [file mmc1.pdf]

Automatic Semantic Segmentation of the Lumbar Spine: Clinical Applicability in a Multi-parametric and Multi-center Study on Magnetic Resonance Images Supplementary Material

Experiment A. Topologies based on the U-Net architecture

In the first experiment, different topologies were designed based on the U-Net architecture with the original U-Net architecture used to obtain baseline results. To do this, a set of distinct interchangeable block types strategically combined to form encoder and decoder branches were defined (see Subsection 4.1).

Table 3 describes the combination of configuration parameters used to obtain optimal results for each network topology, as indicated in Subsection 5.2.

The lumbar spine MR imaging dataset used in this work was extracted from the MIDAS corpus by randomly selecting studies corresponding to 181 patients (see Subsection 3.1.).

Input for neural networks is composed by Sagittal T1w and T2w slices aligned at the pixel level. The ground-truth metadata consists of bit masks generated from the manual segmentation carried out by two expert radiologists with high expertise in skeletal muscle pathology.

All variations designed from the U-Net architecture were trained for 300 epochs using the training subset in the three-fold cross-validation iterations. The optimal version of each model at each cross-validation iteration corresponds to the weight values of the epoch in which the model achieved the highest accuracy with the validation subset.

The reported results were computed after labelling every single pixel to one of the 12 classes with both *Maximum a Posteriori Probability Estimation* (MAP) and *Threshold Optimisation* (TH) (see Subsection 4.3).

Experiment A.1. Results

- Table A.1 and table A.2 shows the Intersection over Union (IoU) per class computed according to (3) and the averaged IoU calculated according to (4) for all the proposed topologies.The results of topologies FCN and U1 are used as a baseline. The averaged IoU including the background class is only shown for informational purposes. The best results for each one of the classes have been highlighted in bold.

  Table A.1 shows the results obtained by using the MAP criterion to label each pixel in one of the target classes. Each pixel in the output is assigned to the class with the highest score generated by the *softmax* activation (see subsection 4.3).

  Table A.2 shows the results obtained by using the TH criterion to label each pixel in one of the target classes. A threshold per target class was tuned using the validation subset to compute the value of the IoU metric for different thresholds (see subsection 4.3).

- Topology UMD obtained the best results of all the variants tested in this work, outperforming the baseline architecture U-Net (U1) for all classes using the two labelling criteria.

- The second best performing topology was UAMD, slightly below UMD, but slightly improves in the *Vertebrae* (IoU_1), *Sacrum* (IoU_2) and *Intervertebral Disc* (IoU_3) classes.

- *Nerve Root* (IoU_9), *Blood Vessels* (IoU_{10}), *Epidural Fat* (IoU_6) and *Intramuscular Fat* (IoU_7) are the most challenging classes to be detected.

- Seven of the proposed topologies (UD, UAD, UMD, UAMD, UDD, UMDD, UDD2) outperforms the two baseline architectures: the standard U-Net and the FCN. Four of these topologies use multi-kernels at the input and Deep Supervision at the output generated by the last level of the decoder branch, or DS.v3.

- The TH labeling criterion performed significantly better than MAP for all experiments.

Experiment B. Ensembles - Model Averaging Technique

For this experiment, the output of several networks corresponding to different topologies is combined to form a classifier that is an ensemble of classifiers using the model averaging technique (see Figure 6).

Model averaging is a technique where R models equally contribute to obtaining the ensemble's output. Two ways of computing the output of the ensemble from the output of the components were considered, the arithmetic mean (Arith) and the geometric mean (Geo) (see Subsection 4.2.1.).

*Corresponding authors:
jsaenz@laberit.com (J.J. Sáenz-Gamboa),
delaiglesia_mar@gva.es (M. de la Iglesia-Vayá)
**https://doi.org/10.1016/j.artmed.2023.102559

Table A.1: **Performance of automatic semantic segmentation via several network topologies. The Maximum A Posteriori Probability Estimate (MAP) criteria were used to label every pixel into one of the target classes (see Subsection 4.3.1.)**. The IoU metric is used to evaluate the performance of the twelve classes using equation (3). The average with/without the background class was computed using equation (4).

**Class**		**Labelling according to the MAP criterion.**												
		**Baseline**		**Variant**										
**#**	**Id**	**FCN**	**U1**	**UA**	**UD**	**UAD**	**UMD**	**UAMD**	**UVMD**	**UVDD**	**UQD**	**UDD**	**UMDD**	**UDD2**
0	**Background**	91.6%	92.2%	92.2%	92.2%	92.2%	92.2%	92.1%	92.2%	92.1%	92.1%	**92.2%**	92.3%	92.2%
1	**Vert**	83.7%	86.0%	85.9%	**86.2%**	86.1%	86.1%	86.1%	86.0%	86.0%	85.7%	86.0%	86.1%	86.1%
2	**Sacrum**	80.8%	84.1%	84.1%	84.4%	84.3%	84.4%	**84.4%**	84.0%	84.0%	83.9%	84.3%	84.3%	84.4%
3	**Int-Disc**	86.4%	88.7%	88.5%	88.8%	88.7%	88.9%	**88.9%**	88.7%	88.7%	88.4%	88.7%	88.7%	88.8%
4	**Spinal-Cavity**	71.9%	75.5%	75.7%	75.6%	75.6%	**75.9%**	75.8%	75.4%	75.6%	75.5%	75.6%	75.6%	75.6%
5	**SCT**	91.7%	92.5%	92.5%	**92.6%**	92.6%	92.6%	92.5%	92.3%	92.4%	92.3%	92.5%	92.5%	92.5%
6	**Epi-Fat**	54.5%	58.0%	58.0%	58.2%	58.3%	**58.5%**	58.3%	57.9%	58.1%	57.4%	58.1%	58.2%	58.1%
7	**IM-Fat**	60.4%	63.8%	63.6%	63.9%	63.8%	**64.2%**	64.0%	63.3%	63.6%	63.0%	63.8%	63.8%	63.9%
8	**Rper-Fat**	69.6%	70.8%	70.6%	**70.9%**	70.84%	70.5%	70.6%	70.6%	70.8%	70.4%	70.7%	70.9%	70.8%
9	**Nerve-Root**	44.9%	50.9%	51.2%	51.2%	51.0%	**51.6%**	51.4%	51.2%	50.7%	50.9%	51.1%	51.3%	51.2%
10	**Blood-Vessels**	57.8%	60.8%	60.4%	61.1%	60.8%	60.9%	60.9%	60.4%	60.8%	60.3%	**61.4%**	61.1%	60.7%
11	**Muscle**	79.0%	80.8%	80.7%	80.9%	80.9%	**81.0%**	80.9%	80.6%	80.8%	80.4%	80.9%	80.9%	80.9%
**IoU** without Bg.		71.0%	73.8%	73.8%	74.0%	73.9%	**74.0%**	74.0%	73.7%	73.8%	73.5%	73.9%	73.9%	73.9%
**IoU** with Bg.		72.7%	75.3%	75.3%	75.5%	75.4%	**75.6%**	75.5%	75.2%	75.3%	75.0%	75.4%	75.5%	75.4%

Table A.2: **Performance of automatic semantic segmentation via several network topologies. The Threshold Optimisation (TH) criteria were used to label every pixel into one of the target classes (see Subsection 4.3.2.)**. The IoU metric is used to evaluate the performance of the twelve classes using equation (3). The average with/without the background class was computed using equation (4).

**Class**		**Labelling according to the TH criterion.**												
		**Baseline**		**Variant**										
**#**	**Id**	**FCN**	**U1**	**UA**	**UD**	**UAD**	**UMD**	**UAMD**	**UVMD**	**UVDD**	**UQD**	**UDD**	**UMDD**	**UDD2**
0	**Background**	91.8%	92.3%	92.3%	92.3%	92.3%	92.2%	92.2%	92.3%	92.22%	92.3%	92.3%	**92.3%**	92.3%
1	**Vert**	84.1%	86.2%	86.2%	86.4%	86.3%	86.3%	**86.5%**	86.3%	86.1%	86.0%	86.2%	86.4%	86.3%
2	**Sacrum**	81.0%	84.3%	84.5%	84.5%	84.6%	84.8%	**84.8%**	84.5%	84.2%	84.1%	84.4%	84.6%	84.6%
3	**Int-Disc**	86.9%	88.9%	88.8%	89.0%	88.9%	89.1%	**89.1%**	89.0%	88.9%	88.8%	88.9%	88.9%	89.0%
4	**Spinal-Cavity**	72.6%	75.8%	76.1%	75.9%	75.8%	**76.1%**	76.1%	75.8%	75.9%	75.7%	75.9%	75.9%	75.9%
5	**SCT**	91.8%	92.6%	92.6%	**92.7%**	92.6%	92.6%	92.5%	92.4%	92.5%	92.4%	92.6%	92.6%	92.5%
6	**Epi-Fat**	54.6%	58.3%	58.3%	58.5%	58.6%	**58.9%**	58.7%	58.3%	58.5%	57.7%	58.5%	58.6%	58.4%
7	**IM-Fat**	61.1%	64.0%	64.0%	64.2%	64.1%	**64.6%**	64.4%	63.7%	64.0%	63.4%	64.1%	64.1%	64.2%
8	**Rper-Fat**	69.3%	70.8%	70.7%	**71.0%**	70.9%	70.6%	70.6%	70.7%	70.8%	70.4%	70.8%	71.0%	70.9%
9	**Nerve-Root**	45.6%	51.8%	51.6%	51.8%	51.7%	**52.3%**	52.1%	51.6%	51.4%	51.3%	51.7%	52.0%	51.8%
10	**Blood-Vessels**	58.7%	61.3%	61.0%	61.4%	61.3%	61.31%	61.3%	60.9%	61.3%	60.9%	**61.7%**	61.4%	61.2%
11	**Muscle**	79.4%	81.1%	81.1%	81.1%	81.2%	**81.2%**	81.1%	80.9%	81.1%	80.8%	81.1%	81.2%	81.1%
**IoU** without Bg.		71.4%	74.1%	74.1%	74.2%	74.2%	**74.3%**	74.3%	74.0%	74.1%	73.8%	74.2%	74.2%	74.2%
**IoU** with Bg.		73.1%	75.6%	75.6%	75.7%	75.7%	**75.8%**	75.8%	75.5%	75.6%	75.3%	75.7%	75.7%	75.7%

In addition to training and evaluating individual semantic segmentation models designed as variations from the U-Net architecture (see Experiment A), a set of ensembles were created by grouping from 4 to 13 models. Table 4 reports all the ensembles used; note that we used the FCN network only in ensembles $E8$ and $E13$ for comparison purposes.

The experiments for each evaluated network topology or ensemble were carried out following the same three-fold cross-validation procedure (See Section 5.).

The proposed network topologies use the *softmax* activation function in the output layer; their outputs are normalized and sum 1. We refer to them as vectors of normalized scores.

The models output masks corresponding to 256×256 patches are combined and generate a single mask per original slide (medical image) to evaluate the quality of the automatic semantic segmentation.

The output of the ensemble is also one vector of normalized scores per pixel. The reported results were computed after labelling each single pixel to one of the 12 classes by either the MAP criterion (see Subsection 4.3.1).

Experiment B.1. Results

- Table B.3 and Table B.4 shows the IoU per class computed according to (3) and the averaged IoU calculated according to (4) for all ensembles using the *Model Averaging* technique, The averaged IoU including the background class is only shown for informational purposes. Two ways of computing the output of the ensemble from the output of the components were used: *Arithmetic mean* (1) (Arith) and *Geometric mean* (2) (Geo), the results shown in B.3

Table B.3: **Performance of automatic semantic segmentation via several ensembles using the Model Averaging technique, computed by using the Arithmetic mean (Arith) (see Equation (1)). The MAP criteria were used to label every pixel into one of the target classes (see Subsection 4.3.1.)**. The IoU metric is used to evaluate the performance of the twelve classes using equation (3). The average with/without the background class was computed using equation (4).

**Class**		**Model Averaging - Ensembles according to Table 5. + Arith + MAP**									
#	**Id**	**E4**	**E5**	**E6**	**E7**	**E8**	**E9**	**E10**	**E11**	**E12**	**E13**
0	**Background**	92.5%	92.5%	92.5%	92.6%	92.6%	92.6%	92.6%	92.6%	92.6%	**92.6%**
1	**Vert**	86.7%	86.8%	86.8%	86.8%	86.8%	86.8%	86.6%	86.8%	**86.8%**	86.8%
2	**Sacrum**	85.1%	85.2%	85.1%	85.2%	85.2%	85.2%	85.2%	85.2%	85.2%	**85.2%**
3	**Int-Disc**	89.2%	89.3%	89.3%	89.3%	89.3%	89.3%	**89.4%**	89.3%	89.36%	89.4%
4	**Spinal-Cavity**	76.6%	76.6%	76.7%	76.8%	76.8%	76.8%	76.8%	76.8%	76.8%	**76.8%**
5	**SCT**	92.9%	93.0%	93.0%	93.0%	93.0%	92.9%	93.0%	93.0%	**93.0%**	93.0%
6	**Epi-Fat**	59.7%	59.7%	59.8%	59.9%	59.9%	60.0%	60.0%	60.0%	60.0%	**60.0%**
7	**IM-Fat**	65.2%	65.3%	65.4%	65.4%	65.4%	65.5%	65.5%	65.5%	**65.5%**	65.5%
8	**Rper-Fat**	71.8%	71.8%	71.9%	71.9%	72.0%	72.0%	72.0%	72.0%	72.0%	**72.0%**
9	**Nerve-Root**	52.8%	52.8%	52.9%	53.0%	53.1%	53.0%	53.1%	53.0%	53.1%	**53.1%**
10	**Blood-Vessels**	62.8%	62.6%	62.8%	62.8%	63.0%	63.0%	63.0%	63.0%	63.0%	**63.0%**
11	**Muscle**	81.7%	81.7%	81.8%	81.8%	81.8%	81.9%	81.9%	81.9%	81.9%	**81.9%**
**IoU** without Bg.		75.0%	75.0%	75.0%	75.1%	75.1%	75.1%	75.1%	75.1%	75.2%	**75.2%**
**IoU** with Bg.		76.4%	76.5%	76.5%	76.5%	76.6%	76.6%	76.6%	76.6%	76.6%	**76.6%**

Table B.4: **Performance of automatic semantic segmentation via several ensembles using the Model Averaging technique, computed by using the Geometric mean (Geo) (see Equation (2)). The MAP criteria were used to label every pixel into one of the target classes (see Subsection 4.3.1.)**. The IoU metric is used to evaluate the performance of the twelve classes using equation (3). The average with/without the background class was computed using equation (4).

**Class**		**Model Averaging - Ensembles according to Table 5. + Geo + MAP**									
#	**Id**	**E4**	**E5**	**E6**	**E7**	**E8**	**E9**	**E10**	**E11**	**E12**	**E13**
0	**Background**	92,5%	92,5%	92,5%	92,6%	92,6%	92,6%	92,6%	92,6%	92,6%	**92,6%**
1	**Vert**	86,7%	86,8%	86,8%	86,9%	86,8%	86,9%	86,9%	86,9%	**86,9%**	86,9%
2	**Sacrum**	85,1%	85,2%	85,2%	85,2%	85,2%	85,2%	85,3%	85,2%	85,3%	**85,3%**
3	**Int-Disc**	89,2%	89,3%	89,3%	89,4%	89,3%	89,4%	**89,4%**	89,4%	89,4%	89,4%
4	**Spinal-Cavity**	76,6%	76,6%	76,7%	76,7%	76,7%	76,7%	76,8%	76,8%	76,8%	**76,8%**
5	**SCT**	92,9%	93,0%	93,0%	93,0%	93,0%	93,0%	93,0%	93,0%	**93,0%**	93,0%
6	**Epi-Fat**	59,7%	59,7%	59,9%	59,9%	59,9%	60,0%	60,0%	60,0%	60,0%	**60,0%**
7	**IM-Fat**	65,2%	65,3%	65,4%	65,4%	65,4%	65,5%	65,5%	65,5%	**65,5%**	65,5%
8	**Rper-Fat**	71,8%	71,8%	71,9%	71,9%	72,0%	72,0%	72,0%	72,0%	72,0%	**72,0%**
9	**Nerve-Root**	52,8%	52,9%	52,9%	53,0%	53,0%	53,0%	53,1%	53,0%	53,1%	**53,1%**
10	**Blood-Vessels**	62,8%	62,6%	62,8%	62,8%	62,8%	63,0%	63,0%	63,0%	63,0%	**63,0%**
11	**Muscle**	81,8%	81,8%	81,8%	81,8%	81,8%	81,9%	81,9%	81,9%	81,9%	**81,9%**
**IoU** without Bg.		75,0%	75,0%	75,0%	75,1%	75,1%	75,1%	75,2%	75,1%	75,2%	**75,2%**
**IoU** with Bg.		76,4%	76,5%	76,5%	76,5%	76,6%	76,6%	76,6%	76,6%	76,6%	**76,6%**

and Table B.4 respectively. MAP criteria were used to label each single pixel into one of thetarget classes. The best results for each one of the classes have been highlighted in bold.

- Ensemble $E13$ obtained the best results of all the ensembles.
- No significant differences between the Arith and the Geo are observed. The high similarity between both ways of computing the mean confirms that all the topologies combined in the ensembles perform very similarly.
- Compared to table A.1 in experiment Experiment A , all ensemble results calculated with the model averaging technique outperforming the baseline architecture U-Net (U1) and the best performing architecture (UMD) in all classes.

Experiment C. Ensembles - Stacking Model Technique

For this experiment, stacking models learn to obtain a better combination of the predictions of R single models to achieve the best prediction.

An ensemble following the stacking model is implemented in three stages: (a) *layer merging*, (b) *meta-learner*, and (c) *prediction*, As indicated in Subsection 4.2.2.

The stacking model technique was used with two different techniquees to prepare the input to the layer-merging stage: (a) the output of the *softmax* activation layer from each model r in

the ensemble, i.e., the vector y_r, and (b) the 64-channel tensor used as input to the classification block (i.e., the outputs generated by the last level of the decoder branch or DS.v3, where applicable). The combination of the inputs in the layer-merging stage can be done by concatenation, averaging, or adding.

The set of ensembles created by grouping 4 to 13 models is shown in table 4.

Ensembles based on the stacking model were trained during 50 epochs using the same data-augmentation transformations used to train each single network (see Subsection 5.4), and following the three-fold cross-validation procedure with the same partitions of the dataset.

Table 5 depicts the best-performing ensemble input formats and layer configurations based on the stacking model assembling technique. A three-letter acronym identifies ensemble configurations. The first letter identifies the input type, **N** and **T** which stand for normalized scores (*softmax* output) and 64-channels tensors, respectively. The second letter indicates layer merging operator: averaging (**A**) and concatenation (**C**). The third corresponds to the type of meta-learner used; in this case, we only used dense layers with the third letter fixed to **D**.

The ensemble configurations are:

– NAD configuration, the inputs to the stacking model are *normalized scores*, the layer-merging is Average, and the meta-learner is a dense layer.

– TCD configuration, the inputs to the stacking model are 64-channel *tensors* at the input of the classification block from each model. The merging layer is a concatenated layer, and the meta-learner is a dense layer.

The stacking model output masks corresponding to 256×256 patches are used to be combined and generate a single mask per original slide (medical image) to evaluate the quality of the automatic semantic segmentation. We use the vector corresponding to each pixel of the reconstructed mask to assign each pixel to one of the twelve classes using MAP or TH (see Subsection 4.3).

Experiment C.1. Results

- Tables C.5, C.6, C.7 and C.8 shows the Intersection over Union (IoU) per class computed according to (3) and the averaged IoU calculated according to (4) for all ensembles using the stacking model technique, The averaged IoU including the background class is only shown for informational purposes.

  The results obtained with the two stacking model configurations and the respective method used to label each pixel in one of the target classes are shown in the tables as follows:

  – NAD + TH in Table C.5, – NAD + MAP in Table C.6

  – TCD + TH in Table C.7, – TCD + MAP in Table C.8

  The best results for each one of the classes have been highlighted in bold.

- The ensemble $E12$+NAD+TH obtained the best overall results. Let us remark that the TH labelling criterion performed better than the MAP criterion in all the performed experiments.

- The ensembles $E10$+TCD+TH and $E12$+NAD+TH performed significantly better than best performing topology tested in this work (UMD+TH) for all target classes.

- The ensembles including the FCN topology, $E8$ and $E13$, have a significant performance drop. Comparing $E12$ and $E13$ results for the configuration NAD+TH, it can be observed that the addition of the FCN topology significantly deteriorates the performance.

Table C.5: **Performance of automatic semantic segmentation via several ensembles using the stacking model assembling technique with NAD configuration (see Subsection 5.4.). The TH criteria were used to label every pixel into one of the target classes (see Subsection 4.3.2.).** The IoU metric is used to evaluate the performance of the twelve classes using equation (3). The average with/without the background class was computed using equation (4).

Class		Stacking Model - Ensembles according to Table 5. + NAD + TH									
#	Id	E4	E5	E6	E7	E8	E9	E10	E11	E12	E13
0	**Background**	92.5%	92.5%	92.6%	92.6%	92.3%	92.6%	92.6%	**92.6%**	92.6%	92.6%
1	**Vert**	86.9%	86.9%	87.0%	87.0%	86.9%	87.0%	**87.0%**	87.0%	87.0%	86.9%
2	**Sacrum**	85.1%	85.1%	85.2%	85.2%	85.2%	85.3%	85.3%	85.4%	**85.4%**	85.2%
3	**Int-Disc**	89.4%	89.4%	89.4%	89.5%	89.4%	89.5%	89.4%	**89.5%**	89.5%	89.4%
4	**Spinal-Cavity**	76.7%	76.7%	76.8%	76.8%	76.3%	76.9%	76.7%	76.8%	**77.0%**	76.7%
5	**SCT**	92.9%	93.0%	93.0%	93.0%	93.0%	93.0%	93.1%	93.0%	93.1%	**93.1%**
6	**Epi-Fat**	59.6%	59.6%	59.8%	59.8%	58.4%	59.9%	**60.0%**	59.9%	60.0%	59.8%
7	**IM-Fat**	65.4%	65.5%	65.5%	65.6%	65.2%	65.6%	65.7%	65.7%	**65.7%**	65.6%
8	**Rper-Fat**	71.6%	71.8%	71.9%	71.9%	**72.3%**	72.0%	72.0%	72.0%	72.0%	72.0%
9	**Nerve-Root**	53.0%	53.0%	53.3%	53.2%	52.2%	53.3%	**53.3%**	53.3%	53.3%	52.2%
10	**Blood-Vessels**	62.8%	62.9%	63.0%	63.0%	63.3%	63.2%	63.2%	**63.4%**	63.3%	63.3%
11	**Muscle**	81.8%	81.9%	81.9%	82.0%	81.8%	82.1%	82.0%	**82.1%**	82.0%	82.0%
**IoU** without Bg.		75.0%	75.1%	75.2%	75.2%	74.9%	75.2%	75.2%	75.3%	**75.3%**	75.1%
**IoU** with Bg.		76.5%	76.5%	76.6%	76.6%	76.3%	76.7%	76.7%	76.7%	**76.7%**	76.6%

Table C.6: **Performance of automatic semantic segmentation via several ensembles using the stacking model assembling technique with NAD configuration (see Subsection 5.4.). The MAP criteria were used to label every pixel into one of the target classes (see Subsection 4.3.1.).** The IoU metric is used to evaluate the performance of the twelve classes using equation (3). The average with/without the background class was computed using equation (4).

Class		Stacking Model - Ensembles according to Table 5. + NAD + MAP									
#	Id	E4	E5	E6	E7	E8	E9	E10	E11	E12	E13
0	**Background**	92.4%	92.5%	92.5%	92.6%	92.3%	92.6%	92.6%	**92.6%**	92.5%	92.5%
1	**Vert**	86.7%	86.9%	87.0%	86.7%	86.8%	**87.0%**	86.9%	86.9%	86.9%	86.9%
2	**Sacrum**	84.9%	84.9%	84.8%	85.1%	84.9%	85.1%	84.9%	85.1%	**85.1%**	85.1%
3	**Int-Disc**	89.2%	89.3%	89.4%	89.3%	89.3%	89.4%	89.4%	**89.4%**	89.3%	89.3%
4	**Spinal-Cavity**	76.4%	76.3%	76.5%	76.5%	75.9%	**76.7%**	76.30%	76.5%	76.5%	76.2%
5	**SCT**	92.9%	93.0%	93.0%	93.0%	92.9%	93.0%	93.0%	93.0%	93.0%	**93.0%**
6	**Epi-Fat**	59.3%	59.3%	59.5%	59.5%	58.0%	59.6%	**59.6%**	59.6%	59.3%	59.4%
7	**IM-Fat**	65.2%	65.3%	65.3%	**65.4%**	65.0%	65.4%	65.4%	65.4%	65.4%	65.4%
8	**Rper-Fat**	71.6%	71.7%	71.8%	71.9%	**72.1%**	71.9%	72.0%	71.9%	71.9%	72.0%
9	**Nerve-Root**	52.6%	52.7%	**53.0%**	52.7%	51.8%	52.8%	53.0%	52.9%	51.8%	51.8%
10	**Blood-Vessels**	62.6%	62.6%	62.8%	62.7%	63.0%	63.0%	62.9%	**63.1%**	62.8%	62.9%
11	**Muscle**	81.7%	81.8%	81.8%	81.8%	81.7%	**81.9%**	81.9%	81.9%	81.9%	81.9%
**IoU** without Bg.		74.8%	74.9%	75.0%	75.0%	74.7%	**75.1%**	75.0%	75.1%	74.9%	74.9%
**IoU** with Bg.		76.3%	76.4%	76.4%	76.4%	76.1%	**76.5%**	76.5%	76.5%	76.4%	76.4%

Table C.7: **Performance of automatic semantic segmentation via several ensembles using the stacking model assembling technique with TCD configuration (see Subsection 5.4.). The TH criteria were used to label every pixel into one of the target classes (see Subsection 4.3.2.)**. The IoU metric is used to evaluate the performance of the twelve classes using equation (3). The average with/without the background class was computed using equation (4).

**Class**		**Stacking Model - Ensembles according to Table 5. + TCD + TH**									
**#**	**Id**	**E4**	**E5**	**E6**	**E7**	**E8**	**E9**	**E10**	**E11**	**E12**	**E13**
0	**Background**	92.4%	92.4%	92.4%	92.4%	92.1%	92.5%	92.5%	**92.4%**	92.4%	92.4%
1	**Vert**	86.6%	86.7%	86.7%	86.7%	86.6%	86.7%	**86.7%**	86.7%	86.7%	86.7%
2	**Sacrum**	85.0%	85.0%	84.8%	85.0%	84.8%	84.9%	**85.0%**	85.0%	85.0%	84.9%
3	**Int-Disc**	89.2%	89.2%	89.2%	89.3%	89.2%	89.2%	**89.3%**	89.2%	89.3%	89.3%
4	**Spinal-Cavity**	76.3%	76.3%	76.4%	76.3%	75.7%	76.3%	**76.5%**	76.4%	76.3%	76.3%
5	**SCT**	92.8%	92.8%	92.8%	92.8%	92.8%	92.9%	92.9%	92.9%	**92.9%**	92.9%
6	**Epi-Fat**	59.2%	59.3%	59.2%	59.2%	57.9%	59.4%	**59.4%**	59.4%	59.2%	59.2%
7	**IM-Fat**	64.8%	64.9%	64.8%	64.9%	64.4%	65.0%	**65.1%**	65.0%	65.0%	65.0%
8	**Rper-Fat**	71.3%	71.5%	71.5%	71.5%	**71.7%**	71.6%	71.6%	71.6%	71.6%	71.6%
9	**Nerve-Root**	52.5%	52.5%	52.3%	52.4%	51.2%	52.3%	**52.6%**	52.3%	51.5%	51.5%
10	**Blood-Vessels**	62.51%	62.3%	62.3%	62.2%	62.2%	**62.7%**	62.6%	62.6%	62.7%	62.7%
11	**Muscle**	81.5%	81.4%	81.5%	81.5%	81.3%	81.6%	81.6%	81.6%	81.6%	**81.7%**
**IoU** without Bg.		74.7%	74.7%	74.7%	74.7%	74.3%	74.8%	**74.8%**	74.8%	74.7%	74.7%
**IoU** with Bg.		76.2%	76.2%	76.2%	76.2%	75.8%	76.2%	**76.3%**	76.3%	76.2%	76.2%

Table C.8: **Performance of automatic semantic segmentation via several ensembles using the stacking model assembling technique with TCD configuration (see Subsection 5.4.). The MAP criteria were used to label every pixel into one of the target classes (see Subsection 4.3.1.)**. The IoU metric is used to evaluate the performance of the twelve classes using equation (3). The average with/without the background class was computed using equation (4).

**Class**		**Stacking Model - Ensembles according to Table 5. + TCD + MAP**									
**#**	**Id**	**E4**	**E5**	**E6**	**E7**	**E8**	**E9**	**E10**	**E11**	**E12**	**E13**
0	**Background**	92.3%	92.4%	92.4%	92.4%	92.0%	**92.4%**	92.4%	92.4%	92.4%	92.4%
1	**Vert**	86.4%	86.5%	86.5%	86.6%	86.4%	86.5%	**86.6%**	86.6%	86.6%	86.5%
2	**Sacrum**	84.8%	**84.8%**	84.8%	84.8%	84.6%	84.8%	84.8%	84.8%	84.8%	84.8%
3	**Int-Disc**	89.0%	89.1%	89.0%	89.1%	89.1%	89.1%	89.1%	89.1%	**89.1%**	89.1%
4	**Spinal-Cavity**	75.9%	76.0%	76.0%	76.0%	75.5%	76.1%	76.1%	**76.1%**	76.0%	76.0%
5	**SCT**	92.7%	92.8%	92.7%	92.8%	92.7%	92.8%	**92.8%**	92.8%	92.8%	92.8%
6	**Epi-Fat**	58.8%	59.0%	58.8%	58.9%	57.6%	58.2%	**59.1%**	58.9%	58.9%	58.9%
7	**IM-Fat**	64.6%	64.6%	64.6%	64.6%	64.2%	64.7%	**64.8%**	64.7%	64.7%	64.7%
8	**Rper-Fat**	71.3%	71.4%	71.4%	71.4%	**71.6%**	71.50%	71.6%	71.5%	71.5%	71.5%
9	**Nerve-Root**	52.0%	51.9%	51.8%	51.8%	50.7%	51.7%	**52.0%**	51.6%	50.9%	51.0%
10	**Blood-Vessels**	62.2%	62.0%	61.9%	61.9%	61.9%	62.3%	62.3%	62.2%	**62.3%**	62.3%
11	**Muscle**	81.3%	81.3%	81.3%	81.3%	81.1%	81.4%	81.4%	81.4%	81.4%	**81.5%**
**IoU** without Bg.		74.5%	74.5%	74.4%	74.5%	74.1%	74.5%	**74.6%**	74.5%	74.5%	74.5%
**IoU** with Bg.		76.0%	76.0%	75.9%	75.7%	75.6%	76.0%	**76.1%**	76.0%	76.0%	75.9%
